# **α**-Actinin TvACTN3 of *Trichomonas vaginalis* Is an RNA-Binding Protein That Could Participate in Its Posttranscriptional Iron Regulatory Mechanism

**DOI:** 10.1155/2014/424767

**Published:** 2014-03-02

**Authors:** Jaeson Santos Calla-Choque, Elisa Elvira Figueroa-Angulo, Leticia Ávila-González, Rossana Arroyo

**Affiliations:** Departamento de Infectómica y Patogénesis Molecular, Centro de Investigación y de Estudios Avanzados del IPN (CINVESTAV-IPN), 07360 México, DF, Mexico

## Abstract

*Trichomonas vaginalis* is a sexually transmitted flagellated protist parasite responsible for trichomoniasis. This parasite is dependent on high levels of iron, favoring its growth and multiplication. Iron also differentially regulates some trichomonad virulence properties by unknown mechanisms. However, there is evidence to support the existence of gene regulatory mechanisms at the transcriptional and posttranscriptional levels that are mediated by iron concentration in *T. vaginalis*. Thus, the goal of this study was to identify an RNA-binding protein in *T. vaginalis* that interacts with the tvcp4 RNA stem-loop structure, which may participate in a posttranscriptional iron regulatory mechanism mediated by RNA-protein interactions. We performed RNA electrophoretic mobility shift assay (REMSA) and supershift, UV cross-linking, Northwestern blot, and western blot (WB) assays using cytoplasmic protein extracts from *T. vaginalis* with the tvcp4 RNA hairpin structure as a probe. We identified a 135-kDa protein isolated by the UV cross-linking assays as **α**-actinin 3 (TvACTN3) by MALDI-TOF-MS that was confirmed by LS-MS/MS and *de novo* sequencing. TvACTN3 is a cytoplasmic protein that specifically binds to hairpin RNA structures from trichomonads and humans when the parasites are grown under iron-depleted conditions. Thus, TvACTN3 could participate in the regulation of gene expression by iron in *T. vaginalis* through a parallel posttranscriptional mechanism similar to that of the IRE/IRP system.

## 1. Introduction

Cellular iron is an essential cofactor for many biochemical activities, including oxygen transport, cellular respiration, and DNA synthesis. Thus, iron deficiency can cause cell growth arrest and death. However, iron overload is also potentially toxic; under aerobic conditions, it catalyzes the formation of reactive oxygen species and generates highly reactive radicals through the Fenton reaction [1]. The dual role of this element has led to the evolution of an elegant regulatory system that maintains iron homeostasis and contributes to its systemic balance [2–4].

In vertebrates, cellular iron homeostasis is maintained by the coordinated expression of proteins involved in iron uptake, storage, utilization, and export, which are regulated at the posttranscriptional level. This mechanism is based on the interactions of cytoplasmic iron regulatory proteins (IRPs) with conserved RNA stem-loop structures known as iron-responsive elements (IREs), which are located in the untranslated regions (UTRs) of specific mRNAs [4–7], under iron-limited conditions. Depending on the location of the RNA hairpin structures at the 5′- or 3′-UTRs of mRNA, the regulatory outcomes of these interactions are (a) the translation inhibition of 5′-UTR IRE-containing mRNAs and (b) the protection and stability of 3′-UTR IRE-containing mRNAs [3].

The IRE/IRP interaction in the 5′-UTR modulates the expression of mRNAs encoding H- and L-ferritin (IRE-fer), ALAS2, m-aconitase, ferroportin, HIF-2**α**, **β**-APP, and **α**-synuclein, which control iron storage, erythroid iron utilization, energy homeostasis, iron efflux, hypoxic responses, and neurological pathways. Conversely, the IRE/IRP interaction in the 3′-UTR stabilizes mRNAs encoding TfR1, DMT1, Cdc14A, and MRCK**α**, which are involved in iron uptake, iron transport, the cell cycle, and cytoskeletal remodeling [7].

There are two cytoplasmic iron regulatory proteins in vertebrates, namely, IRP-1 and IRP-2. IRP-1 (97 kDa) and IRP-2 (105 kDa) share ~57% sequence identity with one another and ~31% sequence identity with mitochondrial aconitase, but only IRP-1 has retained its cytoplasmic aconitase activity [7]. Although IRP-1 and IRP-2 are similar in sequence and structure, their actions are significantly different. IRP-1 contains a [4Fe-4S] cluster, and high iron concentrations prevent IRE-protein interactions [8]. *In vitro* studies have revealed that the iron-sulfur cluster can be disassembled in the presence of oxidizing (NO and H_2_O_2_) and reducing agents, such as *β*-mercaptoethanol, resulting in greater IRE affinity at high iron concentrations [9, 10]. At low iron concentrations, the iron-sulfur cluster is labile and disassembled, and the RNA binding site is exposed at the IRP, enabling RNA-protein interactions [10]. By contrast, IRP-2 binding to IRE structure is not regulated by the assembly and disassembly of an iron-sulfur cluster.

*Trichomonas vaginalis* is a flagellated protist parasite responsible for trichomoniasis, one of the most common nonviral sexually transmitted infections in humans. This protist is dependent on high levels of iron, favoring its growth and multiplication in culture and in the human vagina, where the iron concentration is constantly changing throughout the menstrual cycle. Iron also differentially regulates some trichomonad virulence properties by unknown mechanisms [11, 12]. Knowledge of iron gene expression regulation in *T. vaginalis *is still very limited.

There is evidence to support the existence of gene regulatory mechanisms at the transcriptional and posttranscriptional levels that are mediated by iron concentration in *T. vaginalis*. An iron-responsive promoter and the regulatory proteins that interact with it are involved in the positive transcriptional regulation of ap65-1 gene expression by iron [13], but this system is specific to this particular gene. Moreover, an atypical IRE hairpin structure has been reported in the mRNA of cysteine proteinase 4 (TvCP4) that is upregulated by iron at the posttranscriptional level. This atypical IRE structure specifically interacts with human IRP-1 and also appears to interact with trichomonad cytoplasmic proteins from parasites grown under iron-restricted conditions [11]. However, the specificity and identity of these proteins are unknown because *T. vaginalis* lacks aconitase activity and genes encoding IRP-like proteins. Interestingly, these trichomonad cytoplasmic proteins also specifically interact with human IRE-fer. Taken together, these data suggest the existence of a posttranscriptional iron regulatory mechanism in *T. vaginalis* that is parallel to the typical IRE/IRP system [11, 12].

Therefore, the goal of this work was to identify at least one of the cytoplasmic RNA-binding proteins of *T. vaginalis* that interacts with these IRE structures to provide insight into the posttranscriptional iron regulatory mechanism of this early-evolved protist parasite. Using RNA electrophoretic mobility shift assay (REMSA) and supershift, UV cross-linking, and Northwestern blot (NWB) assays in concert with mass spectrometry (MS) analysis, we identified and characterized the 135-kDa cytoplasmic protein *α*-actinin (TvACTN3), which has the ability to bind to RNA and could participate in the posttranscriptional iron regulatory mechanism of *T. vaginalis*.

## 2. Materials and Methods

### 2.1. Parasite and HeLa Cell Cultures

*T. vaginalis* parasites from a fresh clinical CNCD 147 isolate were cultured in trypticase-yeast extract-maltose (TYM) medium [14] supplemented with 10% (v/v) heat-inactivated horse serum (HIHS) and incubated at 37°C for 24 h. Regular TYM-HIHS medium contains 20 *μ*M iron [14, 15]. Parasites in the logarithmic phase of growth were also cultured in iron-depleted or iron-rich conditions (0 or 250 *μ*M iron, resp.), and the culture medium was supplemented with 150 *μ*M 2,2′-dipyridyl (Sigma-Aldrich, Co., St. Louis, MO, USA) or 250 *μ*M ferrous ammonium sulfate (J. T. Baker, USA) solutions, respectively, 24 h prior to parasite inoculation [11, 15]. HeLa cells were grown in Dulbecco's modified Eagle medium (DMEM) (Invitrogen-Gibco, Carlsbad, CA, USA), supplemented with 10% HIHS at 37°C for 48 h in a 5% CO_2_ atmosphere to obtain confluent cell monolayers [16].

### 2.2. Parasite Cell Fractionation

Parasite cell fractionation was performed by combining previously described methods [17] with a few modifications. In brief, *T. vaginalis *parasites (5 × 10^8^ with a cell density of 1.5 × 10^6^ parasites/mL) cultured under different iron conditions were harvested at 750 ×g for 5 min at 4°C and washed twice with ice-cold PBS, pH 7.0. The parasite pellet was resuspended in 1 mL of extraction buffer (EB) (10 mM HEPES (pH 7.6), 3 mM MgCl_2_, 40 mM KCl, 5% glycerol, and 0.5% Nonidet P-40; Sigma) containing protease inhibitors (7.5 mM TLCK and 1.6 mM leupeptin; Sigma), vortexed, and incubated for 20 min on ice. The parasite lysate was centrifuged for 10 min at 1,000 ×g. The pellet (P1: nucleus and cell debris) was discarded. The supernatant (SN1) was centrifuged for 10 min at 10,000 ×g, and the new supernatant (SN2: cytoplasmic extracts) was stored at −70°C for up to two weeks until use in REMSA and supershift, UV cross-linking, and NWB assays. The pellet (P2: membrane extracts) was resuspended in 750 *μ*L buffer R (20 mM HEPES (pH 7.6), 2 mM MgCl_2_, 1 mM EDTA) and stored at −70°C until use.

### 2.3. Cytoplasmic Extracts from HeLa Cells

HeLa cell cytoplasmic extracts were prepared as previously described [11]. In brief, HeLa cells (40 × 10^6^ with a cell density of 1.5 × 10^6^ cells/mL) were detached with 0.05 M EDTA, for 5 min at 25°C, centrifuged at 2,500 ×g for 5 min at 4°C and washed twice with ice-cold PBS. The cell pellet was resuspended in lysis buffer A (10 mM HEPES (pH 7.9), 15 mM MgCl_2_, and 10 mM KCl), homogenized in a Dounce homogenizer (10–20 strokes), and centrifuged at 10,000 ×g for 30 min at 4°C. The supernatants were diluted to a final protein concentration of 10 mg/mL and stored at −70°C until use.

### 2.4. *In Vitro* Transcription of RNA Sequences

The DNA used for *in vitro* transcription included the following: the plasmid pSPT-fer (kindly donated by Dr. Lukas Kühn), which contains the human ferritin H-chain IRE (IRE-fer) region was linearized with *Bam*HI [18], and two tvcp4 amplicons, namely, (i) amplicon 31 from −3 to 28 nt (including the IRE-tvcp4 sequence) and (ii) amplicon 97 from 12 to 107 nt (a *deletion mutant* that disrupts IRE-tvcp4) [11]. The amplicons were produced by PCR with the primers sense (31), antisense (31), sense (97), and antisense (97) (Table 1). The PCR sense primers contained a bacteriophage T7 promoter sequence (underline nt) and an additional GG sequence to enhance transcription. The purified PCR products (Qiaquick kit, Qiagen Mexico, S. de R.L. de C.V. Mexico) were used as templates for RNA synthesis using an *in vitro* transcription kit (Ambion, Inc. Austin, TX, USA). The transcription reaction was conducted according to the manufacturer's recommendations. Following transcription, the DNA templates were removed by treatment with DNase I (Ambion), and unincorporated nucleotides were removed by precipitation with 5 *μ*g glycogen, 100 mM ammonium acetate salts, and two volumes of absolute ethanol. To synthesize radiolabeled RNA transcripts, 20 *μ*Ci [*α* ^32^P] UTP (800 Ci/mmol; Dupont Mexico, S.A. de C.V., Mexico) was included in the transcription reaction. The IRE-fer transcript encoding the canonical IRE was used as the positive control where indicated. An unrelated RNA transcript was used as a negative control [19]. To obtain the RNA secondary structure, transcripts were incubated at 70°C for 15 min and cooled down at room temperature for 20 min.

### 2.5. RNA Electrophoretic Mobility Shift Assay (REMSA) and Supershift Assay

REMSA assays were performed to detect RNA-protein interactions as reported by Leibold and Munro [20] with some modifications. In brief, 200,000 cpm (10–15 ng) of ^32^P-UTP-labeled RNAs was incubated for 20 min at 4°C with 20 *μ*g cytoplasmic HeLa cell extracts or 50 *μ*g *T. vaginalis* cytoplasmic extracts from parasites grown in iron-rich and iron-depleted conditions in interaction buffer (10 mM HEPES (pH 7.6), 3 mM MgCl_2_, 40 mM KCl, 5% glycerol in DEPC water) in the presence of 20 U RNasin (Roche), 4 *μ*g tRNA, and 2% *β*-mercaptoethanol (only for assessing interactions in the HeLa cytoplasmic extract). After incubation, 20 U RNase T1 and 10 *μ*g RNase A were added to the mixture and incubated for 30 min at 25°C. Then, heparin (5 *μ*g) was added and incubated for 10 min at 4°C. The RNA-protein complexes (RPCs) were resolved on 6% nondenaturing polyacrylamide gels and visualized by autoradiography [18]. To assess specificity, competition REMSAs were performed by adding 50 and 100-fold molar excesses of unlabeled RNA, followed by incubation for 30 min at 4°C. For the supershift assays, 1-2 *μ*L (1 *μ*g/*μ*L) commercial polyclonal antibody against recombinant *α*-actinin from chicken (*α*-chACTN, PA1-28036, Thermo Scientific), a monoclonal antibody against recombinant *α*-actinin from bovine ([BM 75.2], Abcam), 5–15 *μ*L rabbit *α*-TvACTN3r serum, or 10 *μ*L control rabbit against *T. vaginalis* triosephosphate isomerase 2r (*α*-TvTIM2r) serum [21] (as an unrelated antibody) was added, along with the cytoplasmic extracts from trichomonads grown in iron-depleted conditions, before adding the RNA probes. These experiments were independently performed at least three times, with similar results.

### 2.6. UV Cross-Linking Assay

Trichomonad cytoplasmic extracts and the recombinant proteins TvACTN3r, the TvACTN3 domains (DIr, DIIr, and DIIIr), hIRP-1r (used as a positive control), and BSA (1 *μ*g) (used as a negative control) were incubated with 200,000 cpm (10–15 ng) ^32^P-labeled RNA probes for 30 min at 4°C in 25 *μ*L reaction buffer (10 mM HEPES-KOH [pH 7.4], 3 mM MgCl_2_, 5% (v/v) glycerol, 100 mM KCl, 20 U RNasin, and 5 *μ*g yeast tRNA; Invitrogen). After RNA-binding, the reaction mixture was placed on ice and irradiated with a UV-lamp (UVP; 800,000 *μ*J/cm^2^) for 15 min. Unprotected RNA was digested for 30 min at 25°C with RNase A (10 *μ*g) and RNase T1 (20 U). RPCs were resolved on 10% SDS-PAGE gels. The gels were stained with Coomassie Brilliant Blue (CBB) and dried, and radioactive bands were visualized by autoradiography in a FLA 5000 phosphoimager (Fujifilm, Co, Tokyo Japan) with a Software MultiGauge V3.0 [22]. These experiments were independently performed at least three times, with similar results.

### 2.7. Identification of RNA-Binding Proteins in *T. vaginalis*

The high molecular weight protein bands detected by UV cross-linking assays of the cytoplasmic proteins from trichomonads grown under iron-depleted conditions and the IRE-tvcp4 RNA probe were excised from duplicate CBB-stained gels and submitted to protein identification by MS at the Protein Unit of Columbia University (New York, USA). The protein bands were reduced and alkylated, digested with trypsin, and analyzed by matrix-assisted laser desorption ionization time-of flight mass spectrometry (MALDI-TOF-MS), MS/MS and *de novo* sequencing as previously reported [23, 24]. The specific peptides identified (Table 2) corresponded to the TvACTN3 protein. The score and protein coverage were calculated by the MS-Fit MOWSE and Mascot search algorithms.

### 2.8. Cloning and Expression of Recombinant TvACTN3r, DIr, DIIr, and DIIIr Proteins

The complete tvactn3 gene (TVAG_239310; 3390 bp) was amplified by PCR using genomic DNA from the *T. vaginalis* CNCD 147 isolate as a template with a sense primer containing a *Bam*HI restriction site (tvactn3*-Bam*HI) and an antisense primer containing a *Not*I restriction site (tvactn3*-Not*I); these primers included the ATG and TAA initiation and stop codons, respectively (Table 1). The corresponding DNA fragments of Domain I (873 bp, 1–291 aa), Domain II (1713 bp, 230–800 aa), and Domain III (1407 bp, 662–1129 aa) were also amplified by PCR using plasmid DNA (containing the complete tvactn3 gene) with specific primers for each fragment (Table 1). The amplicons were cloned into the pCR4-TOPO vector (Invitrogen). The inserts were released by double digestion and subcloned into the pProEX-HTb expression vector according to the manufacturer's instructions (Invitrogen). The DNA sequence of tvactn3 was deposited in GenBank with accession number KF280188. His-tagged recombinant TvACTN3r, DIr, DIIr, and DIIIr were expressed in *E. coli *BL-21 (DE3) by induction with 1 mM IPTG for 16 h at 16°C. The recombinant proteins were purified from the soluble fraction by affinity chromatography using Ni-NTA-Sepharose as recommended by the manufacturer (GE Healthcare Bio-Sciences Corp, Piscataway NJ, USA). Purified recombinant proteins were dialyzed three times against PBS at 4°C and quantified by the Bradford method (Bio-Rad).

### 2.9. Generation of Anti-TvACTN3r, DIr, DIIr, and DIIIr Antibodies

Female New Zealand white rabbits weighing 3.0 kg were intramuscularly immunized twice with 300 *μ*g affinity-purified TvACTN3r, DIr, DIIr, or DIIIr proteins in a 1 : 1 ratio with TiterMax Gold (Sigma) adjuvant, as recommended by the manufacturer. The animals were bled weekly, and their sera were tested by WB assays against each recombinant protein and cytoplasmic extracts from *T. vaginalis *cultured in regular medium. Before rabbit immunization, the preimmune (PI) serum was obtained from each rabbit and used as a negative control for all experiments with antibodies.

### 2.10. Antibodies

To detect the presence of cross-reacting IRP-1-like proteins in *T. vaginalis*, we used an affinity-purified rabbit polyclonal antibody against rat IRP-1 (rIRP-1, AB15506, Millipore). We also used a rabbit polyclonal antibody against *α*-chACTN. In addition, rabbit polyclonal antisera were produced against purified recombinant TvACTN3r and its domains (*α*-TvACTN3r, *α*-DIr, *α*-DIIr, and *α*-DIIIr). To produce control sera for the western blot (WB) assays of different cellular fractions, we used a mouse polyclonal antibody against a trichomonad PFO A protein for the membrane fraction [23] and a rabbit polyclonal antiserum against a trichomonad cytoplasmic HSP70 protein (produced by Torres-Romero et al., manuscript in preparation) for the cytoplasmic fraction. To produce a negative control serum for the supershift assays, we used a rabbit polyclonal antibody against the recombinant TvTIM2r protein [21].

### 2.11. Western Blotting

After electrophoresis, recombinant TvACTN3r, DIr, DIIr, or DIIIr, or cytoplasmic extracts of *T. vaginalis* grown in 0, 20, or 250 *μ*M iron were transferred onto nitrocellulose (NC) membranes and blocked using 10% fat-free milk in PBS-0.05% Tween 20 (PBS-T) buffer for 18 h at 4°C. The NC membranes were washed five times with PBS-T at 25°C and incubated for 18 h at 4°C with each antibody. The antibodies were diluted in PBS-T as follows: *α*-TvACTN3r (1 : 150,000), *α*-DIr (1 : 1,000), *α*-DIIr (1 : 1,000), and *α*-DIIIr (1 : 1,000). The NC membranes were washed five times with PBS-T at 25°C, incubated with secondary antibodies (*α*-rabbit or *α*-mouse immunoglobulin Gs [IgGs] coupled to peroxidase) (Bio-Rad) at a 1 : 3,000 dilution in 10% fat-free milk in PBS-T for 2 h at 25°C, washed five times with PBS-T, and developed with 4-chloro-1-naphthol (Bio-Rad) or by chemiluminescence using a SuperSignal West Pico Kit (Pierce). The corresponding PI rabbit or mouse serum was used as a negative control. These experiments were independently performed at least three times and yielded similar results.

### 2.12. Northwestern Blotting (NWB)

The Northwestern blot assay was performed by combining previously described methods [17, 24] with a few modifications. In brief, recombinant proteins were resolved by 10% SDS-PAGE, transferred onto NC membranes, blocked with 10% fat-free milk and 20 *μ*g/mL yeast tRNA (Sigma) in EB (EBY) for 18 h at 4°C, and washed three times with EBY at 25°C. The NC membranes were incubated for 18 h at 4°C with EBY containing 10–15 ng/mL radiolabeled RNA; washed twice with EB; washed another two times with B buffer (10 mM Tris-HCl [pH 7.5] and 50 mM NaCl); and blocked again for 1 h at 37°C with STMT buffer (1 M NaCl, 0.1 M Tris-HCl [pH 7.5], 2 mM MgCl_2_, and 0.05% Triton X-100) containing 3% BSA. After extensive washing with EB, the NC membranes were air-dried and exposed for autoradiography. These experiments were performed independently at least three times, with similar results.

### 2.13. *In Silico* Analysis of TvACTN3

Multiple alignments were performed with the ClustalW program. The nucleotide sequences of all tvactn genes were obtained from the *T. vaginalis* genome project database (http://www.trichdb.org/). To identify functional RNA-binding domains, we analyzed the deduced protein sequence of TvACTN3 using the SMART (http://smart.embl-heidelberg.de/) [26, 27], Motif Scan (http://hits.isb-sib.ch/cgi-bin/PFSCAN), and PROSITE programs (http://prosite.expasy.org/prosite.html) [28].

### 2.14. RNA Isolation, Semiquantitative RT-PCR, and Real-Time qRT-PCR Analyses

The total RNA from parasites (10^7^) grown in 0, 20, or 250 *μ*M iron was extracted using TRIzol reagent (Sigma). For semiquantitative RT-PCR, the total RNA (1 *μ*g) was reverse-transcribed using the SuperScript II reverse transcriptase kit (Invitrogen) and an oligo (dT18) primer. A 0.5 *μ*g quantity of cDNA was then used as a PCR template [25]. Gene-specific primers [29] were used for PCR amplification of the *T. vaginalis α*-actinin (tvactn1, tvactn2, tvactn3, tvactn4, and tvactn5) transcripts (Table 1). To produce an internal control, a 112-bp fragment of the **β**-tubulin gene was amplified by PCR with specific primers [25]. As additional controls, the tvpfo a (iron-upregulated gene) [23] and tvcp12 (iron-downregulated gene) transcripts were also amplified using specific primers [11] (Table 1).

Quantitative RT-PCR was performed for each tvactn gene using an ABIPRISM 7300 Sequence Detector (Applied Biosystems). In brief, the RT-PCR amplification mixtures (25 *μ*L) contained 1 *μ*g cDNA from each condition; 10 pmol specific primers for tvactn1, tvactn2, tvactn3, tvactn4, or tvactn5 (Table 1); and 2× SYBR Green 1 PCR Master Mix (12.5 *μ*L) buffer (Applied Biosystems). The cycling conditions for all tvactn genes and transcripts included 10 min of polymerase activation at 95°C, followed by 25 cycles of 95°C for 20 s, 55°C for 30 s, and 72°C for 30 s. Each assay (in triplicate) included a standard curve of eight 1/10 serial dilutions of tvactn3 or **β*-tub* cDNA (1 *μ*g of each tested cDNA) and a no-template control. All PCR efficiencies were greater than 95%. The results obtained by the Sequence Detection Software (version 1.3; Applied Biosystems) were exported as tab-delimited text and imported into Microsoft Excel for further analysis. To confirm the amplification specificity, PCR products were subjected to standard curve analysis. The levels of tvactn1, tvactn2, tvactn3, tvactn4, or tvactn5 mRNA were quantified by qRT-PCR analysis relative to *β*-tubulin mRNA as an internal control.

### 2.15. Statistical Analysis

All data were expressed as the means ± S.D. from three samples, and qRT-PCR experiments were repeated three times. The significance of the difference between means was determined by one-way ANOVA using Sigma-Plot11. The level of significance was also determined by the Bonferroni method comparing all groups versus control (*P* < 0.001) for Figure 4(b). The scores showing statistical significance are indicated in the figures with asterisks.

### 2.16. Ethical Statement

This study was performed with strict accordance with the recommendations of the Guide for the Use of Laboratory Animals of the Center of Research and Advanced Studies of the National Polytechnic Institute (CINVESTAV-IPN). The protocols and experiments were approved by the Institutional Animal Care “CICUAL” at the CINVESTAV-IPN. Animals were kept in environmentally controlled animal facilities at CINVESTAV-IPN. The final bleeding was performed under sodium pentobarbital anesthesia and efforts were always made to minimize suffering.

## 3. Results

### 3.1. Cytoplasmic Proteins from *T. vaginalis* Grown in Iron-Depleted Concentrations Specifically Interact with IRE-tvcp4 and IRE-fer

**Figure 1:**
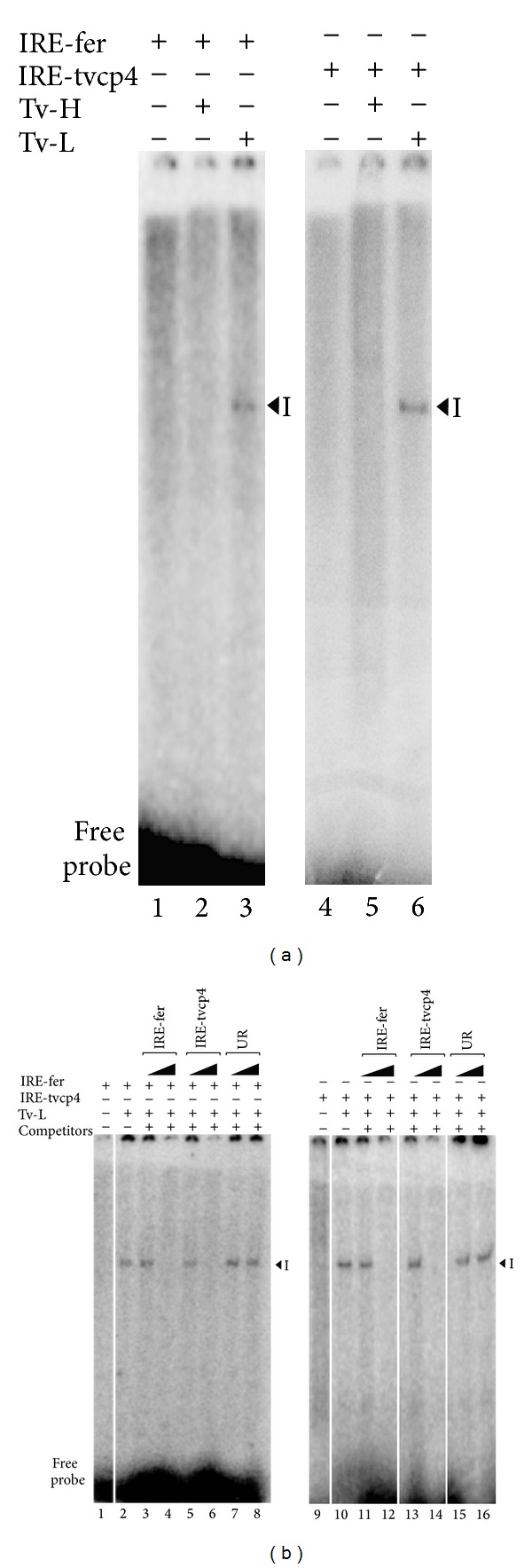
Presence of RNA-binding proteins in iron-depleted *T. vaginalis.* (a) RNA gel-shift assays (REMSA) to detect RNA-protein complex (RPC) formation using IRE-fer (lanes 1–3) and IRE-tvcp4 (lanes 4–6) RNA probes and *T. vaginalis* cytoplasmic extracts from parasites grown in iron-rich (H) (lanes 2 and 3) or iron-depleted (L) (lanes 5 and 6) medium. Experiments with IRE-fer are controls. RNA free probes used as negative controls (lanes 1 and 4). (b) REMSA competition assays performed with the IRE-fer and IRE-tvcp4 RNA probes and *T. vaginalis* cytoplasmic extracts in the absence of competitors (lanes 2 and 10) or in the presence of a 50- or 100-fold molar excess of unlabeled IRE-fer RNA (lanes 3 and 4), IRE-tvcp4 (lanes 13 and 14), or unrelated transcript (lanes 7, 8 and 15, 16). A cross-competition assay in the presence of a 50- or 100-fold molar excess of unlabeled IRE-tvcp4 RNA (lanes 5 and 6) and IRE-fer RNA (lanes 11 and 12). Experiments were performed three times and yielded similar results.

We previously demonstrated the specific interaction between an atypical IRE stem-loop structure in the 5′ region of tvcp4 mRNA with human recombinant IRP and HeLa cell cytoplasmic proteins, which suggested the presence of RNA-binding proteins in trichomonad cytoplasmic extracts [11]. To determine if *T. vaginalis* has RNA-binding proteins that specifically interact with IRE structures and may participate in a posttranscriptional regulatory mechanism parallel to the IRE-IRP system described in other organisms, we performed REMSA with cytoplasmic extracts from *T. vaginalis* grown in iron-rich (Tv-H) and iron-depleted (Tv-L) media and radiolabeled RNA probes of trichomonad IRE-tvcp4, and the human IRE-fer that was used as a positive control.  Figure 1(a) shows the formation of one RNA-protein complex (RPC) with *T. vaginalis* cytoplasmic extracts under iron-depleted conditions using both probes (lanes 3 and 6); the complexes were sensitive to reducing agents (data not shown). These results suggest that there are RNA-binding proteins in the trichomonad extracts that interact with hairpin RNA structures in iron-depleted conditions and in the absence of reducing agents.

RNA competition assays were also performed to determine the specificity of the RPC formed between the trichomonad cytoplasmic proteins and both tested IREs. The addition of a molar excess of unlabeled homologous or heterologous IRE probe (IRE-fer and IRE-tvcp4) relative to labeled IRE significantly decreased the amount of RPC (Figure 1(b), lanes 3–6 and 11–14, resp.) in a concentration-dependent manner. An excess of unlabeled unrelated transcripts was used as competitor RNA [19], which did not affect RPC formation, as expected (Figure 1(b), lanes 7, 8 and 15, 16, resp.). These data demonstrate that the RNA-protein complexes detected with trichomonad cytoplasmic proteins are specific and imply the existence of RNA-binding proteins in *T. vaginalis*, in spite of the lack of aconitase/IRP proteins in trichomonads.

### 3.2. IRE-tvcp4 and IRE-fer mRNA Probes Specifically Interact with at Least Four Protein Bands from *T. vaginalis* Extracts

**Figure 2:**
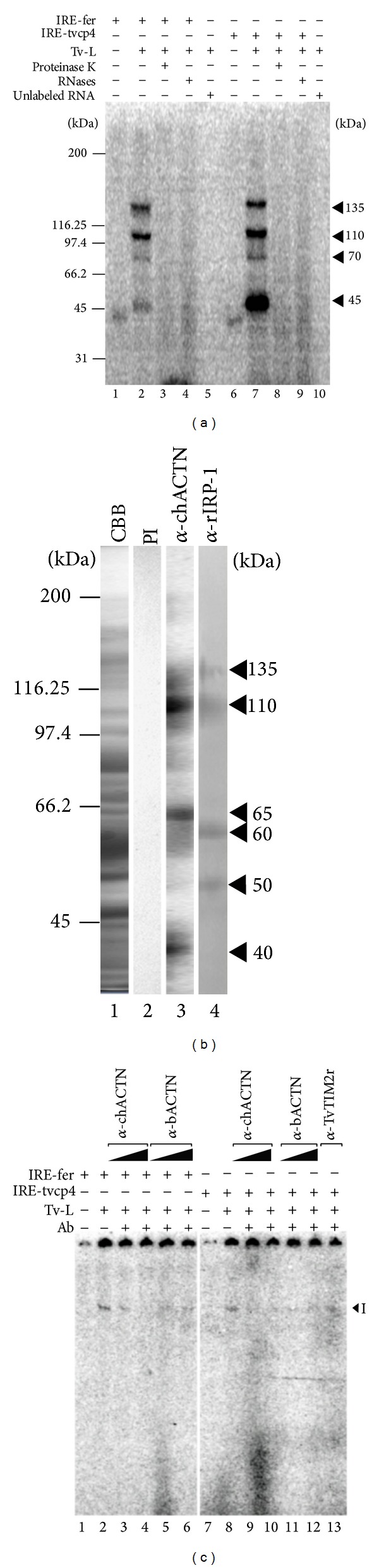
Identification of *T. vaginalis* TvACTN3 by 1-D gel electrophoresis and mass spectrometry. (a) UV Cross-linking assay of ^32^P-labeled IRE-fer and IRE-tvcp4 transcripts and *T. vaginalis* cytoplasmic extracts grown under iron-depleted conditions (Tv-L) (lanes 2 and 7, resp.). The specificity of the interaction was demonstrated by treatment with proteinase K (10 *μ*g, Promega) (lanes 3 and 8) and RNases (lanes 4 and 9) and a complete binding reaction using unlabeled IRE-fer and IRE-tvcp4 (lanes 5 and 10) or only the labeled probes (lanes 1 and 6). Molecular mass markers are indicated in kilodaltons (kDa). Arrowheads indicate the positions of the RNA-protein complex bands of 135, 110, 70, and 45 kDa. A representative result of three independent experiments with similar results is shown. (b) Cytoplasmic extracts of *T. vaginalis* grown in iron-depleted medium were Coomassie blue-stained (lane 1) or transferred onto NC membranes for Western blot (WB) assays (lanes 2, 3, and 4); and incubated with preimmune (PI) serum (negative control, lane 2), anti-chicken *α*-actinin (*α*-chACTN, lane 3), or anti-rat-IRP-1 (*α*-IRP1, lane 4) antibodies. (c) Representative supershift assays. Radiolabeled IRE-fer and IRE-tvcp4 RNA probes (lanes 1 and 7, resp.) were incubated with cytoplasmic proteins from *T. vaginalis* grown under iron-depleted conditions without (lanes 2 and 8) or with the *α*-chACTN polyclonal antibody (1 *μ*g) (lanes 3 and 9), (2 *μ*g) (lanes 4 and 10), or with the anti-bACTN monoclonal antibody (1 *μ*g) (lanes 5 and 11), (2 *μ*g) (lanes 6 and 12), or with a nonrelated antibody (*α*-TvTIM2r; lane 13). The arrowhead indicates RNA-protein complex. A representative result of three independent experiments yielding similar results is shown.

To determine the size of the proteins in the RPC, UV cross-linking assays were performed using cytoplasmic extracts from *T. vaginalis *grown in iron-depleted conditions and radiolabeled IRE-tvcp4 and IRE-fer mRNA probes.  Figure 2(a) shows that both RNA probes interacted with at least four proteins of 135, 110, 70, and 45 kDa (lanes 2 and 7). The 45-kDa protein band was more intense in the presence of IRE-tvcp4 than in the presence of IRE-fer. The presence of reactive bands required the simultaneous occurrence of RNA and protein molecules during UV irradiation. Few or no reactive bands were observed when the cross-linking reaction was treated with proteinase K (lanes 3 and 8) or RNases (lanes 4 and 9) or when unlabeled IREs were used (lanes 5 and 10), indicating that the radioactive bands were formed when cytoplasmic trichomonad proteins and labeled-RNA were present in the reaction and specifically interacted with one another.

### 3.3. The 135-kDa Protein Band That Binds to IRE-tvcp4 and IRE-fer Is a *T. vaginalis α*-Actinin (TvACTN3)

To identify the 135-kDa protein detected in the UV cross-linking assays (Figure 2(a)), the corresponding protein band was excised from the duplicate CBB-stained gel and prepared for MS analysis by tryptic mapping of the 135-kDa protein band by MALDI-TOF-MS, MS/MS and *de novo* sequencing. The masses of 35 peptides obtained from the 135-kDa protein band corresponded to peptide masses from the TVAG_239310 cytoplasmic TvACTN3 of *T. vaginalis* [29]. Only 1/35 peptides (peptide 33) was common to TvACTN1 and 2/35 peptides (peptides 4 and 34) were common to TvACTN2 (Table 2). None of the identified peptides were found in TvACTN4 and TvACTN5. The protein sequence coverage was 43% with a MS-Fit MOWSE score: 2.64*e* + 11; Mascot score: 212, and expected value 5.8*e* − 15. To confirm this identification, the 135-kDa protein band was processed by ESI-LC-MS/MS. A single peptide: FMIEEISVEEATAR (peptide 4) was identified. This peptide was common to *α*-actinin TvACTN2 and TvACTN3 of *T. vaginalis*. Since only one peptide was obtained, a new analysis by *de novo* sequencing was performed and four peptide sequences: EEYNQAAQK, TAIAAEK, QECLDVINTER, and IQPTLEEPYQ that corresponded to TvACTN3 of *T. vaginalis* were obtained. Two of them were part of the sequence of peptides 18 and 23 identified by MALDI TOF (Table 2).

To confirm this identification, we performed a WB assay using a commercial polyclonal antibody against recombinant chicken *α*-actinin (*α*-chACTN) with cytoplasmic extracts from iron-depleted *T. vaginalis*. This antibody recognized protein bands of 135, 110, 65, and 40 kDa (Figure 2(b), lane 3). Similar molecular weight protein bands (135, 110, 60, and 50 kDa) were also detected with a commercial polyclonal *α*-rIRP-1 antibody (Figure 2(b), lane 4). Interestingly, these two antibodies showed cross-reactivity to the 135- and 110-kDa protein bands of *T. vaginalis*.

### 3.4. Actinin Proteins from *T. vaginalis* Are Present in the RNA-Protein Complex Formed between Cytoplasmic Extracts and RNA Probes from Humans and Trichomonads

To corroborate the presence of actinin in the RPC formed between trichomonad cytoplasmic proteins and RNA probes (IRE-tvcp4 and IRE-fer), a supershift assay was performed by adding the *α*-chACTN polyclonal antibody and the *α*-bACTN monoclonal antibody to the REMSA reaction.  Figure 2(c) shows that both heterologous anti-ACTN polyclonal and monoclonal antibodies reduced RPC formation between the cytoplasmic extracts of iron-depleted *T. vaginalis* and both IRE probes (lanes 3–6 and 9–12, resp.) at two antibody concentrations; an unrelated antibody was used as a negative control (lane 13). These results confirm the presence of trichomonad *α*-actinins in the RPC.

### 3.5. Only Three of the Five *α*-Actinin Encoding Genes Are Expressed in *T. vaginalis* under Different Iron Concentrations

**Figure 3:**
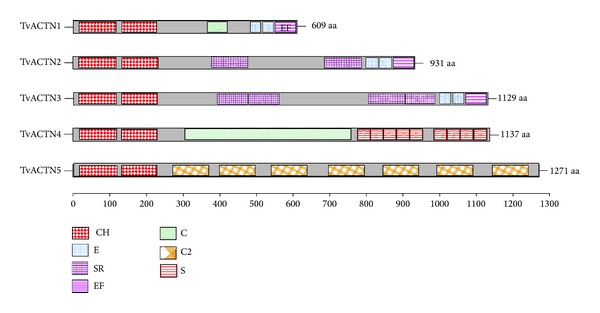
Putative functional domains present in TvACTNs of *T. vaginalis*. Principal motifs of the deduced amino acid sequences of the five tvactn genes reported in the *T. vaginalis* genome sequence [29]. TvACTN1 (609 aa), TvACTN2 (931 aa), TvACTN3 (1129 aa), TvACTN4 (1137 aa), and TvACTN5 (1271 aa). By SMART, MOTIF SCAN, and PROSITE programs, we found the principal motif sequences present in the five TvACTNs proteins, two calponin domains (CH) at the N-terminus followed by variable spectrin repeats (SR) in the central rod domain, and two EF-hands: EF-hand (E) and calcium-insensible EF-hand (EF) at the C-terminus. TvACTN4 and TvACTN5 present more divergent central region and C-terminus. TvACTN4 has one coiled coil (C) and nine SEL domains (S), and TvACTN5 presents seven C2 domains (C2).

Five annotated sequences in the *T. vaginalis *genome encode *α*-actinin genes: TVAG_156680, TVAG_190450, TVAG_239310, TVAG_247460, and TVAG_260390; we named these genes tvactn1, tvactn2, tvactn3, tvactn4, and tvactn5, respectively. We performed a multiple alignment analysis of the deduced amino acid sequences [29] (see Supplementary Figure S1 in Supplementary Material available online at http://dx.doi.org/10.1155/2014/424767). The five *α*-actinin proteins have different sizes (609, 931, 1129, 1137, and 1271 amino acid [aa] residues, resp.) and different identities compared with TvACTN3 (55.19, 53.25, 22.1, and 15.56% with TvACTN1, TvACTN2, TvACTN4, and TvACTN5, resp.). All of the proteins have two calponin domains (CH) at the N-terminus that confer the ability to bind actin, followed by spectrin repeats (SR) in the central rod domain and two EF-hands (EF-H) at the C-terminus. The number of spectrin repeats is variable in TvACTN2 and TvACTN3, and the central domain of TvACTN1, TvACTN4, and TvACTN5 has different specific domains compared to the other *α*-actinins (Figure 3).

**Figure 4:**
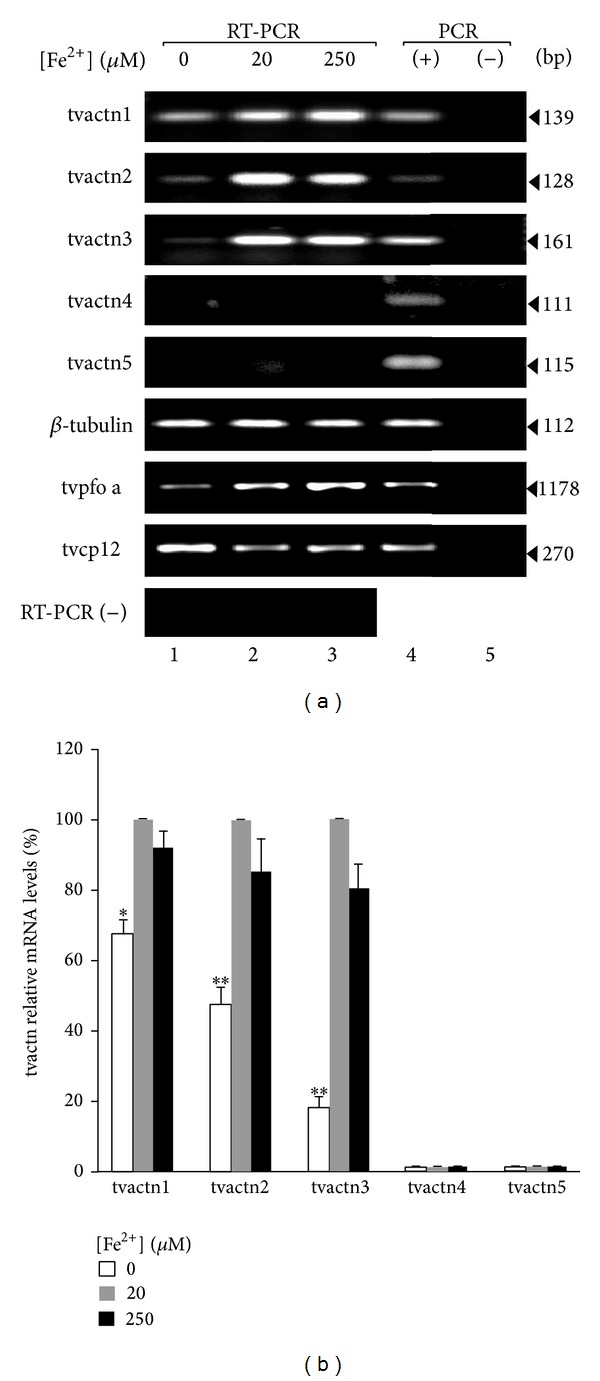
Effects of iron on the transcription of *T. vaginalis* actinin genes. (a) RT-PCR with specific primers for each *T. vaginalis* actinin gene (tvactn1, tvactn2, tvactn3, tvactn4, and tvactn5; Table 1) using cDNA from parasites grown under iron-depleted (0 *μ*M; lane 1), normal (20 *μ*M; lane 2), and iron-rich (250 *μ*M; lane 3) conditions (tvactn1 to tvactn5). RT-PCR with specific primers for the *β*-tubulin gene using the same cDNA as an internal control. tvpfo a and tvcp12 genes were used as control genes that are overexpressed under iron-rich or iron-depleted conditions, respectively. A reverse transcriptase minus [RT-PCR (−)] reaction was used to verify the lack of gDNA contamination in the cDNA samples using the same pairs of primers for each gene; gDNA was used as a positive control (+), except in RT-PCR (−), in which no DNA was added. PCRs without gDNA were used as negative controls (−). The sizes of the amplicons are given in bp. (b) qRT-PCR was used to quantify the different levels of the five tvactn mRNAs in trichomonads grown under different iron conditions. Bars represent the standard error of triplicated samples. Asterisks (*) *P* < 0.005 or (**) *P* < 0.0001 compared iron-depleted with iron-rich or normal conditions.

We then analyzed which of the *T. vaginalis* actn genes are expressed under different iron concentrations. Semiquantitative RT-PCR and qRT-PCR assays using specific primers for each tvactn gene (Table 1) were performed using RNA isolated from trichomonads grown in 0, 20, or 250 *μ*M iron.  Figure 4(a) shows that the *T. vaginalis* tvactn1, tvactn2, and tvactn3 genes were better expressed in the presence of iron (lanes 2 and 3) than under iron-depleted conditions (lane 1). Neither tvactn4 nor tvactn5 was expressed under these experimental conditions (lanes 1–3). The expected control tvactn1–5 gene fragments were amplified from genomic DNA. The level of the *β*-tubulin transcript in the same RNA samples at each iron concentration was used to normalize the transcript quantities. Two genes differentially regulated by iron were also used as controls (tvpfo a for positiveand tvcp12 for negative iron regulation, resp.), and an RT-PCR without reverse transcriptase was used to exclude genomic contamination (Figure 4(a)). The qRT-PCR analysis confirmed these results and demonstrated that the expression of the tvactn3 gene is greatly reduced in the absence of iron in comparison to tvactn2 and tvactn1 expression, and tvactn4 and tvactn5 were not amplified under any of the tested iron concentrations and used experimental conditions. The differences observed among the tvactn1, tvactn2, and tvactn3 amplicons were significant (*P* < 0.0001), with the exception of tvactn1 (*P* < 0.005), and were dependent on the iron concentration (Figure 4(b)).

### 3.6. Cloning and Expression of TvACTN3 and Its Three Domains

**Figure 5:**
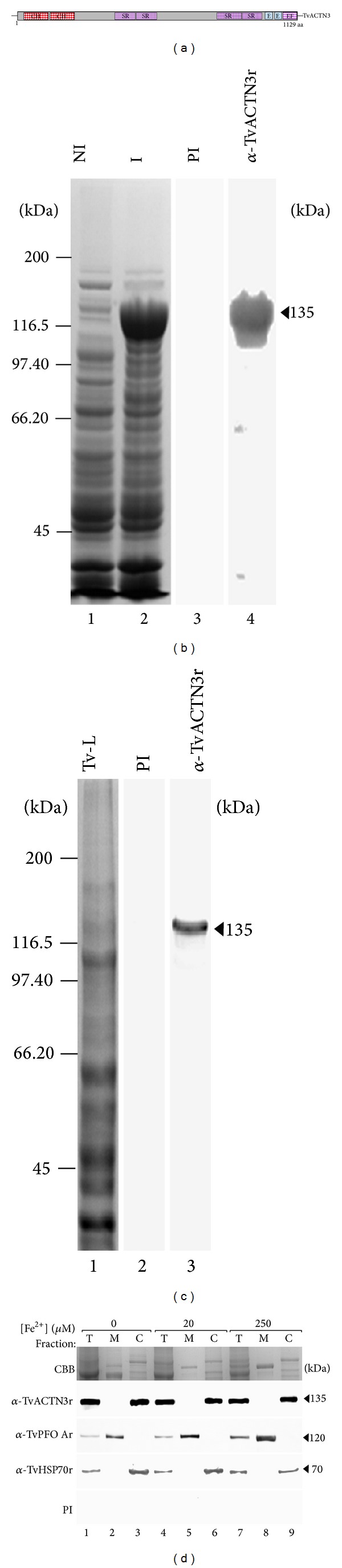
Expression of the native *T. vaginalis* tvactn3 gene and the cloning and expression of recombinant TvACTN3 (TvACTN3r) and its cellular distribution. (a) Principal motifs of the deduced amino acid sequence of the TvACTN3 protein, including Domain I (DI), which contains two calponin-homology domains (CH), followed by four spectrin repeats (SR) in the central rod domain, and three EF-hands (E); one of them is insensitive to calcium (EF) at the C-terminus. (b) Expression of the 6x-His-tagged TvACTN3r protein. Bacteria *E. coli* were transformed with pProEX-HTb-TvACTN3 plasmid and protein expression was induced by the addition of 1 mM IPTG for 16 h at 16°C. Protein extracts were separated through 7.5% SDS-PAGE and gels were stained with Coomassie Brilliant Blue (CBB), noninduced bacterial extract (lane 1), IPTG-induced bacterial extract (lane 2). Immunodetection of TvACTN3r polypeptide by WB assays using specific rabbit antibodies: preimmune (PI) serum used as a negative control (lane 3), *α*-TvACTN3r serum (lane 4). (c) Detection of the native TvACTN3 expression by WB with an *α*-TvACTN3r antibody. CBB-stained *T. vaginalis* total extract (lane 1), *T. vaginalis* total extract transferred onto a NC membrane and incubated with the PI serum (lane 2), or with *α*-TvACTN3r antibody (lane 3). (d) Trichomonad cell fractionation of parasites grown in iron-depleted (0 *μ*M; lanes 1–3), iron-normal (20 *μ*M; lanes 4–6), and iron-rich (250 *μ*M; lanes 7–9) conditions: Total (T), Membrane (M), and Cytoplasmic (C) fractions were analyzed by WB with *α*-TvACTN3r, *α*-TvPFO Ar, and *α*-TvHSP70r polyclonal antibodies. The last two antibodies were used as controls. Arrowheads indicate the size or position of the protein bands detected by antibodies. kDa, molecular weight standards in kilodaltons. A representative result of three independent experiments with similar results.

To demonstrate that the 135-kDa actinin (TvACTN3) is one of the RNA-binding proteins that interacts with the human IRE-fer and the atypical IRE-tvcp4 of *T. vaginalis*, we first performed an *in silico* analysis of the reported tvactn3 gene sequence in the *T. vaginalis *genome (TrichDB Accession no. TVAG_239310). This gene contains a 3390-bp open reading frame (ORF) encoding a complete TvACTN3 protein of 1129 aa residues with a theoretical size ~124.2-kDa and *pI* of 4.9. It has a short 5′-UTR with two putative Inr sequences 3-bp and 10-bp upstream of the ATG initiation codon. The 3′-UTR is 11 bp long and has the typical regulatory regions for polyadenylation [30, 31]. The TvACTN3 protein contains three domains: Domain I (DI), which includes two calponin or actin-binding domains (CH) at the N-terminus; Domain II (DII), which is followed by four spectrin repeats (SR) in the central rod domain; and Domain III (DIII), which includes three EF-hands (EF-H) at the C-terminus (Figure 5(a)).

**Figure 6:**
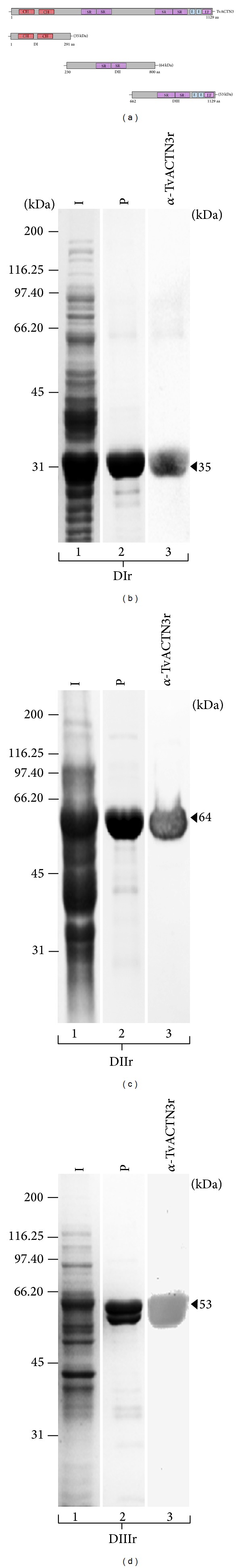
Expression and recognition of TvACTN3 domains DIr, DIIr, and DIIIr by *α*-TvACTN3r antibody. (a) Map of domains used to generate the recombinant proteins (DIr, DIIr, and DIIIr) of TvACTN3. ((b)–(d)) Induction (I), purification (P), and recognition by WB of DIr, DIIr, and DIIIr proteins. Expression of the 6x-His tagged DIr, DIIr, and DIIIr proteins. Bacteria *E. coli* were transformed with pProEX-HTb- DIr, DIIr, or DIIIr plasmid and protein expression was induced by the addition of 1 mM IPTG for 16 h at 16°C. Protein extracts were separated through 10% SDS-PAGE and gels were CBB-stained, IPTG-induced (I) bacterial extract (lanes 1). Affinity purified recombinant proteins using Ni-NTA-Sepharose (lanes 2; P). Immunodetection of DI, DII, or DIII polypeptide by WB assays using *α*-TvACTN3r serum (lanes 3). kDa, molecular weight markers in kilodaltons (Bio-Rad).

We cloned, expressed, and purified the complete tvactn3 gene sequence and its three domains to obtain recombinant proteins for functional assays and to identify the putative RNA-binding domain. The amplicons were obtained by PCR using *T. vaginalis* genomic DNA or plasmid DNA-TvACTN3 and specific primers for each gene fragment (Table 1). These segments were cloned, sequenced, and expressed in a bacterial system after the induction with IPTG (Figures 5 and 6).

The complete TvACTN3 protein was expressed as a recombinant ~135-kDa protein (TvACTN3r) and purified by Ni-affinity chromatography. Polyclonal antibodies against the purified protein were produced in rabbits (*α*-TvACTN3r) (Figure 5(b)). This antibody reacted with a 135-kDa protein band in the total protein extract of parasites grown in iron-depleted conditions; PI serum was used as a negative control (Figure 5(c)). Following cell fractionation of parasites grown in different iron concentrations, this protein was detected in the total extracts and cytoplasmic fractions as expected. The amount of protein was not affected by the iron concentration (Figure 5(d)) compared to the control proteins (PFO A for the membrane and HSP70 for cytoplasmic fractions).

**Figure 7:**
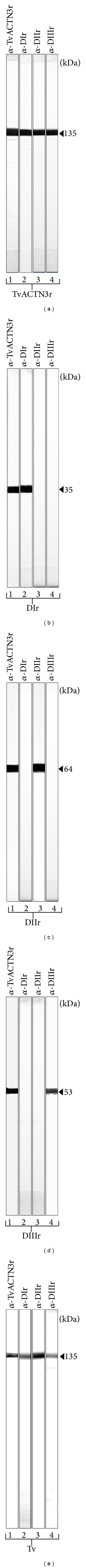
Production of polyclonal antibodies and specific recognition of TvACTN3 domains (DIr, DIIr, and DIIIr) and *T. vaginalis* cytoplasmic proteins by the specific antibodies. WB assays using (a) TvACTN3r; (b) DIr, (c) DIIr, (d) DIIIr purified recombinant proteins; and (e) cytoplasmic proteins from *T. vaginalis *grown in regular iron conditions used as antigens and transferred onto NC membranes and incubated with *α*-TvACTN3r, *α*-DIr, *α*-DIIr, and *α*-DIIIr polyclonal antibodies (lanes 1–4). kDa, molecular weight markers in kilodaltons (Bio-Rad).

We also obtained recombinant proteins and polyclonal antibodies corresponding to each TvACTN3r domain as follows: Actin Binding Domain (DI), Spectrin Repeats Domain (DII), and the EF-hand Domain (DIII), which had molecular weights of 35, 64, and 53 kDa, respectively (Figure 6). Each of the antibodies recognized the complete TvACTN3r protein as well as the native TvACTN3 protein in total protein extracts, as expected. In addition, the antibodies against each domain only recognized the corresponding recombinant protein, while the anti-TvACTN3r antibody recognized all three domains as expected (Figure 7).

### 3.7. The Polyclonal TvACTN3r Antibody Supershifted RNA-Protein Complex I

**Figure 8:**
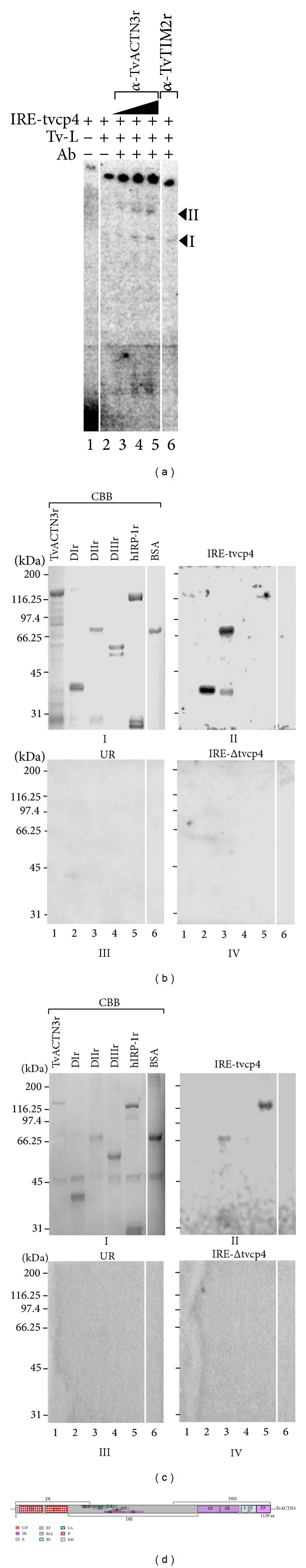
Interaction of TvACTN3r with trichomonad IRE RNA. (a) Representative *α*-TvACTN3r antibody supershift assays. A radiolabeled IRE-tvcp4 RNA probe (lane 1) was incubated with cytoplasmic proteins from *T. vaginalis* grown under iron-depleted conditions without (lane 2) or with different quantities of *α*-TvACTN3r antibody (lanes 3–5) or an unrelated antibody (*α*-TvTIM2r; lane 6). Arrowheads indicate the RNA-protein complexes. A representative result of three independent experiments with similar results is shown. (b) Northwestern blot assay. The recombinant proteins TvACTN3r; DIr, DIIr, and DIIIr and the controls hIRP-1r; and BSA were separated by SDS-PAGE on 10% polyacrylamide gels (panel I) and transferred onto NC membranes, which were incubated with a radiolabeled IRE-tvcp4 RNA probe (panel II). The controls included a radiolabeled nonrelated RNA (panel III) and a radiolabeled *deletion mutant* that disrupts IRE-tvcp4 RNA (panel IV). (c) UV cross-linking assays of the recombinant proteins TvACTN3r, DIr, DIIr, and DIIIr and radiolabeled IRE-tvcp4 RNA probes resolved by SDS-PAGE on 10% polyacrylamide gels (panel II). As controls, TvACTN3r and its domains were incubated with radiolabeled nonrelated RNA (panel III) and a radiolabeled *deletion mutant* that disrupted IRE-tvcp4 RNA (panel IV). In addition, a control from a representative gel of the different recombinant proteins in cross-linking mix was CBB-stained (panel I); kDa, molecular weight standards in kilodaltons. A representative result of three independent experiments with similar results is shown. (d) Identification of putative RNA-protein interaction motifs in TvACTN3 by *in silico* analysis. The complete protein and the different domains of TvACTN3 are illustrated with brackets, and the possible motifs for RNA-protein interactions are indicated. The boxes show the location of the motif described on top of the figure and in Table 3.

To test our hypothesis about the participation of TvACTN3 in RNA-protein interactions, we performed a supershift assay using the *α*-TvACTN3r antibody in an REMSA reaction between trichomonad iron-depleted cytoplasmic extracts and the IRE-tvcp4 RNA probe.  Figure 8(a) shows that the RPC I (lane 2) was supershifted by the *α*-TvACTN3r antiserum in a concentration-dependent manner, and an additional second RPC (II) was also observed (lanes 3–5). Moreover, antibodies against each TvACTN3-specific domain (DIr, DIIr, DIIIr) yielded a similar effect (data not shown). An unrelated negative control antibody at the highest concentration (*α*-TvTIM2r) had no effect on RPC formation, as expected (lane 6). Similar results were obtained by supershift assays when the IRE-fer RNA probe was used in the presence of *α*-TvACTN3r antibody (data not shown). These results showed the participation of TvACTN3 in the ribonucleoprotein complex that binds the two IREs.

### 3.8. Identification of the Putative RNA-Binding Domain(s) of TvACTN3

To identify the putative RNA-binding domain in TvACTN3, we conducted two different functional assays, that is, an NWB assay and a UV cross-linking assay using the purified recombinant proteins (TvACTN3r, DIr, DIIr, and DIIIr).  Figure 8(b) shows that the radiolabeled IRE-tvcp4 RNA probe in the NWB assays specifically bound to the complete recombinant protein (TvACTN3r) and its DIr and DIIr domains as well as the recombinant hIRP-1r, which was used as a positive control. The probe did not bind to the negative control protein BSA (panel II). An absence of RNA-protein interactions was observed when an unrelated radiolabeled RNA and a tvcp4* deletion mutant* that disrupts the IRE-tvcp4 RNA [11] were used as negative control probes (panels III and IV, resp.).

To confirm these results, we also performed UV cross-linking assays to analyze the RNA-protein interactions using the same recombinant proteins and radiolabeled RNA probes as described in Figure 8(b).  Figure 8(c) shows that a specific interaction was only observed between the IRE-tvcp4 RNA probe and the DIIr domain of TvACTN3 (panel II) and hIRP-1r, compared with the negative controls (panels III and IV). Similar results were observed when the radiolabeled IRE-fer RNA probe was used as a positive control (data not shown). Taken together, these results suggest that in trichomonads grown under iron-depleted conditions TvACTN3r may function as an RNA-binding protein using its central domain to interact with IRE mRNA structures.

### 3.9. Domain II of TvACTN3r Has Putative RNA-Binding Domains That May Be Used to Interact with RNA

An *in silico* analysis of DI and DII sequences using the SMART, Motif Scan, and PROSITE programs resulted in the identification of different regions of TvACTN3 DII putative motifs that have been reported to be involved in RNA-protein interactions, such as the BRIGHT (292–386 aa) [32], B5 (344–401 aa) [33], LA (392–448 aa) [34], Pumilio-binding (428–464 aa) [35–37], and KH (433–502 aa) [36–38] domains (Figure 8(d), Table 3). Based on the sequences found in the *T. vaginalis* genome and NCBI database, none of these putative RNA-binding motifs were identified in the other TvACTN proteins (Figure 3; Supplementary Figure S1) or in ACTN from other organisms such as *Aedes aegypti *(gi*|*157115648*|*)*, Gallus gallus *(gi*|*211083*|*)*, Homo sapiens *(gi*|*178058*|*)*, Drosophila melanogaster *(gi*|*22831541*|*)*, Naegleria gruberi *(gi*|*284087152*|*)*, Paracoccidioides braziliensis *(gi*|*226286950*|*)*, Wuchereria bancrofti *(gi*|*402588131*|*), and* Entamoeba dispar *(gi*|*167384828*|*) (data not shown).

## 4. Discussion

Iron plays an important role in host-parasite interactions by stimulating cytoadherence and complement resistance and by reducing cytotoxicity and apoptosis induction in host cells [39]. The mechanisms by which iron modulates virulence gene expression in trichomonads are poorly understood. At the transcriptional level, gene expression is mediated by an iron-responsive promoter [13] and the coordinated interactions of at least three Myb proteins [40–42]. To date, this type of regulation has been described only for the ap65-1 gene, which is upregulated by iron [13]. Recently, the positive expression of TvCP4 by iron was described at the protein level, and predictions of an atypical IRE stem-loop structure located at the 5′ end of the tvcp4 mRNA suggest that an ancestral form of RNA stem-loop structures is involved in a novel iron posttranscriptional regulatory mechanism mediated by RNA-protein interactions parallel to the IRE/IRP system [11, 12].

In this work, we identified and characterized a 135-kDa cytoplasmic protein corresponding to *α*-actinin, called TvACTN3, as one of the protein components involved in RNA-protein interactions that could mediate posttranscriptional regulation by iron in *T. vaginalis*. Our data suggest that this protein is part of a ribonucleomultiprotein complex capable of interacting with the IRE-tvcp4 and IRE-fer stem-loop structures (Figures 2(a), 2(c), and 8; Table 2).

We demonstrated a specific interaction between cytoplasmic proteins in *T. vaginalis* grown under iron-depleted conditions and IRE structures from this parasite as well as human ferritin by REMSA assays (Figure 1). These data support our previous results, suggesting the presence of a posttranscriptional regulatory system in *T. vaginalis* that has been conserved during evolution [11, 12]. However, this parallel mechanism in *T. vaginalis* uses atypical hairpin RNA structures and different RNA-binding proteins (RBPs). This finding is not surprising because this parasite lacks aconitase activity and IRP-like protein encoding genes [29]. Herein, we showed the presence of four cytoplasmic RBPs that specifically bind to IRE-fer and IRE-tvcp4 with molecular weights ranging from 135-kDa to 45-kDa in iron-depleted trichomonad cytoplasmic extracts (Figure 2(a)). This specific localization suggests that these proteins could be working as RBPs. However, the difference in molecular weight relative to IRP-1 or IRP-2, as well as the negative effect of *β*-mercaptoethanol on RNA-protein complex formation, suggests the presence of atypical RBPs in this particular mechanism. Similar results have been reported in other protist parasites [43–47].

Why is actinin 3 one of the RNA-binding proteins in this parasite? According to the genome sequence of *T. vaginalis*, this parasite has five different *α*-actinin-encoding genes [29]. Sequence multiple alignments at the nucleotide and protein levels revealed that the N- and C-terminal domains (Domains I and III) are conserved among the five trichomonad *α*-actinins. The central region (Domain II) is the most divergent due to differences in the number of spectrin repeats in each isoform (Figure 3). However, only one *α*-actinin has been studied previously. The 110-kDa *α*-actinin (dubbed TvACTN2 in here) was found throughout the cytoplasm of pear-shape *T. vaginalis*. In the amoeboid form, high levels of *α*-actinin were found in the periphery of this cell, mainly in the pseudopodia and adhesion plaques that colocalized with the actin protein [48]. In addition, overexpression of tvactn2 gene has been reported for parasites adhered to vaginal epithelial cells and under low iron conditions [49]. Moreover, in the TrichDB database tvactn2 showed the highest number of ESTs obtained from parasites grown under different environmental conditions as compared to the other four tvactn genes. However, the tvactn2 gene showed the lowest number of ESTs under low iron conditions. Our results are consistent with those observed in the TrichDB ESTs (Figure 4), showing less expression of tvactn genes in parasites grown under iron-depleted than under regular or unsynchronized cultures as well as regarding the absence or very poor expression of tvactn4 and tvactn5 genes under different growth conditions.

The participation of TvACTN3 in the RNA-protein complex formation was evaluated by different functional assays utilizing tools we generated, namely, recombinant proteins (TvACTN3r and its three domains) and specific antibodies against each of the recombinant proteins (Figures 5–7). Supershift, NWB, and UV cross-linking assays revealed that the TvACTN3r protein possible through the DIIr domain can bind RNA hairpin structures (Figure 8). These results suggest the role of TvACTN3 as an RNA-binding protein.

The typical role of *α*-actinin is to serve as a ubiquitous cytoskeletal conserved protein in eukaryotic cells that cross-links actin filaments and plays an important role in motion and morphological changes [50]. *α*-Actinin belongs to the spectrin superfamily, which is characterized by the ability to bind actin and by the presence of several spectrin repeats [51]. Its other important roles in the cell are linking the cytoskeleton to different transmembrane proteins, regulating the activity of several receptors and serving as a scaffold to connect the cytoskeleton to diverse signaling pathways. Over the course of evolution, alternative splicing and gene duplication have led to a substantial functional assortment within the *α*-actinin protein family. This diversity is most marked in mammalian cells, in which four *α*-actinin-encoding genes produce at least six different products or isoforms. Each isoform has different tissue and subcellular localizations, expression profiles, and biochemical characteristics [52]. Similar relationships are likely to be expected for the five actinin-encoding genes of *T. vaginalis*, which appear to have specific expression levels under different iron concentrations (Figure 4). These results are consistent with recently reported RT-PCR, microarray, and EST data (http://www.trichdb.org/) [49, 53].

Moreover, the *T. vaginalis* genome has undergone multiple gene duplication events; the presence of five *α*-actinin coding genes likely represents a neofunctionalization process. Although actinin primarily function as an actin-binding protein, gene duplication throughout evolution led to the acquisition of new roles inside the cell, including RNA-binding, depending on the microenvironmental conditions. This is not surprising because cytoskeletal proteins are also involved in RNA-cytoskeletal associations and mRNA localization in most of cell types. The association of RNAs with cytoskeletal proteins is required in cellular processes such as mRNA transport and protein synthesis [54]. Cytoskeletal proteins such as *β*-actin and annexin A2 also contribute to the posttranscriptional regulation of gene expression of specific genes through interactions between these proteins and *cis*-acting elements, generating a higher-order structure and regulating the localization and translation of these specific transcripts [55–57].

Because RNA-protein interactions were identified only under iron-depleted concentrations, we analyzed the expression of the tvactn3 gene. We observed that this gene is upregulated by iron at the mRNA level (Figure 4); however, protein expression appears to be constitutive (Figure 5(d)). The interaction of actinin TvACTN3 with RNA could occur in the absence of iron through some of the putative RNA-binding motifs found in the central DII domain, particularly in the region between residues 230 and 662, as suggested by *in silico* analysis (Figure 8; Table 3). These data suggest that iron may control the interaction of TvACTN3 with actin or RNA possible by causing conformational changes by switching functions depending on the iron concentration but without modifying the amount of protein. However, we can speculate that (a) under iron-rich concentrations TvACTN3 is an actin-binding protein that promotes polymerization and stabilizes actin filaments and that (b) under iron-depleted concentrations TvACTN3 is an RNA-binding protein that interacts with RNA hairpin structures that may be involved in a parallel posttranscriptional iron regulation mechanism. Work is in progress to analyze these possibilities.

The RNA-TvACTN3 interaction seem to be absent in the other four *T. vaginalisα*-actinins and in *α*-actinins from other species when using different computational programs. Thus to identify the putative RNA-binding domain in the TvACTN3 central domain DII, additional studies are needed to define the particular region(s) of DII responsible for the RNA-protein interaction, which could include the putative motifs identified previously (Table 3) or a new uncharacterized motif that might be responsible for the novel RNA-binding property identified in the TvACTN3 of this early evolving protist parasite. It will also be necessary to determine whether TvACTN3 also interacts with other IRE-like structures present in the multiple iron-regulated genes, as recently described [49, 50], in addition to the structure identified in the 3′-UTR region of the iron-downregulated tvcp12 gene, which encodes another CP of *T. vaginalis* and plays a role in cytotoxicity (unpublished results). Thus, this finding could represent a novel posttranscriptional iron regulatory mechanism common to iron-regulated genes in *T. vaginalis*.

## 5. Conclusion

In summary, our results showed that TvACTN3 is one of the cytoplasmic proteins acting as an RNA-binding protein that could participate in iron regulation at the posttranscriptional level by a mechanism mediated by RNA-protein interactions parallel to the IRE/IRP system.

## Supplementary Material

In this figure we show a multiple alignment of the amino acid deduced sequence of the five actinin genes found in the *T. vaginalis* genome [8] (TvACTN1, TvACTN2, TvACTN3, TvACTN4, and TvACTN5). These *α*-actinin proteins have different sizes (609, 931, 1129, 1137, and 1271 amino acid [aa] residues) and different identities compared with TvACTN3 (55.19, 53.25, 22.1, and 15.56% with TvACTN1, TvACTN2, TvACTN4, and TvACTN5, respectively). The most conserved region among them is the N-terminal corresponding to the actin-binding domain. The most divergent region is the central domain that contains the spectrin repeats (Please see Figure 3).

## Figures and Tables

**Table 1:** Primers used for PCR, RT-PCR, and qRT-PCR assays to amplify the distinct tvactn gene fragments found in the draft of the *T. vaginalis* genome sequence, mRNA of each tvactn gene and genes used as controls, complete tvactn3 gene and its three domains for expression, and the different amplicons of the IRE sequences used as RNA probes.

Gene	Location^a^ (bp)	Primers (5′-3′)	
		Forward	Reverse
Tvactn1^b^	1512–1648	CCGCTTGCCTTACAGCACTCGG	CCCTTGTTGAAGTGGTCGAGCAT
Tvactn2^b^	1551–1676	TACACAGTTCCTCGCCAAGCAG	CCTCTGTTGGGCTGTGGTTGACA
Tvactn3^b^	1540–1698	CAGCGCGCTGCCAACGTTGAC	CCAAGGCAAGCTGTGTACATGGCTG
Tvactn4^b^	1561–1667	CCTTAAAGCAACAGTTAGCAGCAGC	CCTGTTCGTTTGCTCTTGCAATTAC
Tvactn5^b^	1561–1673	CCCCAGGCGAAGAACCAGAAATT	CCGTTTGAGTCCATTGCGACTAACT
*β*-Tubulin^b^	611–722	CATTGATAACGAAGCTCTTTACGAT	GCATGTTGTGCCGGACATAACCAT [25]
Tvactn3^c^	1–3390	GGCGGGATCCATGTCTAATAATCGTGGACTTCTAGAC	GGCGGCGGCCGCTTAGGCATAGATAGAATTGAC
Tvactn3 DI^d^	1–873	GGCGGGATCCATGTCTAATAATCGTGGACTTCTAGAC	GGCGAAGCTTTCCTGGAACTGTCTGGTCGTAC
Tvactn3 DII^e^	688–2400	GGCGGGATCCCACTTCTTCGCTGGCGAGTCA	GGCGGCGGCCGCGACCTTCTCGGCGATGGCAAGAACC
Tvactn3 DIII^f^	1984–3390	GGCGGGATCCGACATCACATTCGCCTTCTTGACAC	GGCGGCGGCCGCTTAGGCATAGATAGAATTGAC
Tvpfo a^g^	2281–3458	GAAGAGGGCAAGAACTGGGATC	ATCTTCTTGTAGCCCTCGTAA [23]
Tvcp12^h^	8–276	GATTTCAAACTTGCTTCCGGCATT	CTTGACTGTTTGGCCCTTGGAAA [25]
IRE-tvcp4^i^	−3–28	TAATACGACTCACTATAGGGCACATGTTCGTTCAGGCACCAT	CTTTCTGCTCATGTGCCTGAACGAACATGTG [11]
disrupted IRE-tvcp4^j^	12–107	TAATACGACTCACTATAGGGGGCACATGAGCAGAAAGC	GGAGAGCCAAATGCCAAG [11]

^a^Location of the region amplified by PCR, RT-PCR, and qRT-PCR of each tvactn gene. ^b^Primers used for RT-PCR and qRT-PCR assays to check Tvactn gene expression under distinct iron concentrations. ^c^Restriction sites (underlined) for *Bam*HI and *Not*I enzymes used to subclone the complete tvactn3 gene from the *T. vaginalis* CNCD 147 isolate. ^d^Restriction sites (underlined) for *Bam*HI and *Hind*III enzymes used to subclone the Actin-binding domain (DI) of tvactn3 in a bacterial expression system. ^e^Restriction sites (underlined) for *Bam*HI and *Not*I enzymes used to subclone the Spectrin Repeats Domain (DII) of tvactn3 in a bacterial expression system. ^f^Restriction sites (underlined) for *Bam*HI and *Not*I enzymes used to subclone the EF-hand Domain (DIII) of Tvactn3 in a bacterial expression system. ^g^Primers used for RT-PCR assays to check pfo a gene expression under distinct iron concentrations used as an expression control. ^h^Primers used for RT-PCR assays to check tvcp12 gene expression under distinct iron concentrations used as an expression control. ^i^Primers used for PCR of IRE-tvcp4 sequences. ^j^Primers used for PCR of a *deletion  mutant* that disrupts the IRE-tvcp4.

**Table 2:** Peptides identified by MALDI-TOF-MS analysis of the 135-kDa protein band of *T. vaginalis* that interacted with RNA IRE-tvcp4 probe by UV cross-linking assay (Figure 2(a))^a^.

Peptide number^b^	Measured *m*/*z* (av)^c^	Calculated *m*/*z* (av)^d^	Error^e^	Position^f^	Peptide sequences (aa)^g^
1	1516.59	1516.65	−0.06	6–18	(R)GLLDDAWEQTQIK(V)
2	1749.93	1749.91	0.02	32–47	(K)GIPFDNVLEEFADGVK(L)
3	1640.95	1641.02	−0.07	48–61	(K)LIQLLEIVSKEPMK(G)
4	1624.73	1624.81	−0.08	119–132	(K)FMIEEISVEEATAR(D)
5	928.98	929.11	−0.13	133–140	(R)DALLLWAK(K)
6	2099.52	2099.28	0.24	171–187	(K)FRPNMLDYDSLDQTQQK(E)
7	2114.98	2115.28	−0.30	171–187	(K)FRPNMLDYDSLDQTQQK(E) + Oxidation (M)
8	2030.39	2030.24	0.15	220–237	(K)SVVTQVAEFFHFFAGESK(T)
9	1308.21	1308.39	−0.18	254–264	(K)AIEEEALNYEK(Q)
10	1369.87	1369.63	0.24	294–304	(K)SKLFNCIKFGR(V)
11	1180.53	1180.44	0.09	305–314	(R)VVRPVIVDKR(G)
12	1279.12	1279.48	−0.36	340–350	(K)EELLPPNLNLK(F)
13	1392.58	1392.51	0.07	398–409	(K)AINLTGDLYEQR(D)
14	1934.50	1934.07	0.43	410–427	(R)DALNNYLQQAQEAAGTVK(E)
15	1599.90	1599.83	0.08	428–440	(K)ELQPQFVELVELR(L)
16	1847.28	1847.11	0.17	448–464	(R)TVIAVDGEFEQLIATIK(R)
17	2003.26	2003.30	−0.04	448–465	(R)TVIAVDGEFEQLIATIKR(L)
18	1321.37	1321.44	−0.06	482–492	(K)KIEEYNQAAQK(Y)
19	1214.35	1214.33	0.03	502–512	(K)QDLEAIAGELR(E)
20	1762.73	1762.98	−0.25	530–544	(R)NGVSDIRPMFQELEK(Q)
21	1779.30	1778.98	0.32	530–544	(R)NGVSDIRPMFQELEK(Q) + Oxidation (M)
22	3063.49	3063.38	0.12	545–572	(K)QSLHLGIENTPDAVTAMYTACLSQAQDK(I)
23	2108.58	2108.35	0.24	628–645	(K)ASIQPTLEEPYQYLQSIK(Y)
24	3199.79	3199.39	0.40	660–687	(R)DSDITFAFLTTLLNQLEEQLQSESNDAR(I)
25	1363.80	1363.47	0.33	698–708	(K)YVDIANEFHQK(V)
26	1732.90	1732.94	−0.04	720–734	(R)RNAYLSAQLELGNKR(E)
27	1576.51	1576.75	−0.24	721–734	(R)NAYLSAQLELGNKR(E)
28	3097.45	3097.48	−0.03	750–777	(R)DTLHIRVNDSPATISKVYANALQIITDK(L)
29	1656.07	1655.93	0.14	802–816	(K)VVQNVELTGTLLELK(D)
30	3332.99	3332.65	0.34	824–851	(K)AQAQEILPELPTLDAPWEDLCDFNLNYR(V)
31	1549.64	1549.68	−0.04	992–1004	(K)GLQISEEQLTEFR(E)
32	2601.90	2601.78	0.12	1005–1024	(R)ETFNHFDKDHTNFLQYFELR(A)
33	1027.15	1027.13	0.03	1054–1061	(K)LNFDEYVK(F)
34	1024.19	1024.19	0.00	1062–1069	(K)FMLDHFSK(A)
35	3225.53	3225.52	0.01	1082–1109	(K)AIANNNPILTDAQLDQYFKGEEAEYLRK(V)

^a^Masses listed represent 43% sequence coverage with 2.64*e* + 11 MS-Fit MOWSE score and 212 Mascot score and 5.8*e* − 15 expect value. ^b^Consecutive number assigned to the identified peptides. ^c^Measured peptide mass average [*m*/*z* (av)] obtained by MALDI-TOF-MS after tryptic digestion of the 135-kDa protein band excised from a duplicate CBB-stained gel used as a control for the UV cross-linking assays (Figure 2(a)). This protein was identified as actinin3 from *T. vaginalis* (tvactn3, TVAG239310). ^d^Calculated peptide mass average [*m*/*z* (av)] obtained from a theoretical tryptic digestion of the deduced amino acid sequence of the *T. vaginalis  * tvactn3 gene (TVAG 239310; tvactn3) reported in the genome of* T. vaginalis* [29]. ^e^Error represents the difference after comparing the measured and calculated peptide mass averages [*m*/*z* (av)]. ^e^Position in amino acid residues of the identified peptides (start-end) in the deduced amino acid sequence of *T. vaginalis* tvactn3 gene, tvactn3. ^g^Amino acid sequence of the peptides obtained from a theoretical tryptic digestion of tvactn3 (see Figure S1).

**Table 3:** Putative motifs present in tvactn3 that may be involved in DNA-RNA interactions.

Domain	Sequence^a^	Consensus sequence/60%^b^	Location aa*	GO Function	Reference
BRIGHT/ARID	VKSKLFNCIKFGRVVRPVIVDKRGVAMKTWGQ LVTKCNSNGRPIPQVKEELLPPTLNLKFEEIEKT ASDRRDELTKILEELQAKLINAFDEAANA	pppp.FbppLbpFbccptp...b.plPhls .cs..lDLapLa.hVpcbGGbppVsps+p. Wpclsppbsbss.......sssthpL+pbYb+bLbsaEpbbp.	292–386	DNA-binding	[32]

B5	PPTLNLKFEEIEKTASDRRDELTKILEELQAKLI NAFDEAANAKIAVCDDINNKAINL	.pslplphsclscllGhsls.....pclhclLpcLG.... bplp.ps.....s.bpVplPsaR..hDlpbc....hDLlEEltRlaGY	344–401	RNA-binding, Magnesium ion binding, ATP binding	[33]

LA	DDINNKAINLTGDLYEQRDALNNYLQQAQEAA GTVKELQPQFVELVELRLNNRVKRT	pclppplb+QlEaYFScbNLspDbFLpcpbs c..-GaVPlphlssFp+l+ sL∗p-....hpbIspAL.+p S.....sblcls-c...sp+ lRRp..	392–448	RNA-binding	[34]

Pumilio-Binding	ELQPQFVELVELRLNNRVKRTVIAVDGEFEQLIATIK	.bpsplhplsp.cpaGspllQ+hlcbss.p... pppbllp...	428–464	RNA-binding	[35–37]

KH	FVELVELRLNNRVKRTVIAVDGEFEQLIATIKRL IEGNKAAIFEYENKKKIEEYNQAAQKYVDEVAQLKQ	..s.hpbpl..lss....pb htblIG+pG. ..pslcplppps.ssplpl. p.............pphlpl pts....pslpbsbpbl.pblp....	433–502	RNA binding domain	[36–38]

^a^The protein sequence of tvactn3 was used to search in the SMART 7 program to identify the presence of DNA-RNA binding putative domains in the aa sequence. The computational program used for this analysis presented the aa in a single word code. The other codes mean the following: (−) aa negatively charged: D or E; (*) aa S or T; (l) Aliphatic aa: I, L, V; (+) aa positive charged; (t) A, G, S aa; (a) aa with aromatic group: F, H, W, Y; (c) charged aa: D, E, H, K, R; (s) small aa: A, C, D, G, N, P, S, T, V; (p) polar aa: C, D, E, H, K, N, Q, R, S, T; (b) Big aa: E, F, H, I, K, L, M, Q, R, W, Y; (h) Hydrophobic aa: A, C, F, G, H, I, L, M, T, V, W, Y. ^b^A PSI-BLAST search using the complete tvactn3 sequence (residues 1–1129) of *T. vaginalis* and *E* < 0.01 enable us to identify each of the DNA-RNA binding domains sequences (BRIGHT/ARID, B5, LA, Pumilio-Binding, and KH domains) present in the DII of tvactn3 shown in Figure 8(d). These aa sequences showed a 60% similitude with the consensus sequences of these motifs by multiple alignments.
