# Corrigendum to “*α*-Actinin TvACTN3 of *Trichomonas vaginalis* Is an RNA-Binding Protein That Could Participate in Its Posttranscriptional Iron Regulatory Mechanism”

**DOI:** 10.1155/2016/2676598

**Published:** 2016-07-19

**Authors:** Jaeson Santos Calla-Choque, Elisa Elvira Figueroa-Angulo, Leticia Ávila-González, Rossana Arroyo

**Affiliations:** Departamento de Infectómica y Patogénesis Molecular, Centro de Investigación y de Estudios Avanzados del IPN (CINVESTAV-IPN), 07360 México, DF, Mexico

 In Figures 7(a)–7(d) of the published article titled “*α*-Actinin TvACTN3 of* Trichomonas vaginalis* Is an RNA-Binding Protein That Could Participate in Its Posttranscriptional Iron Regulatory Mechanism” [1], we mistakenly used the same strip data in panel (a), lanes 3 and 4; panel (b), lanes 3 and 4; panel (c), lanes 2 and 4; and panel (d), lanes 2 and 3. The corrected new Figure 7 and legend are presented here.

## Figures and Tables

**Figure 7:**
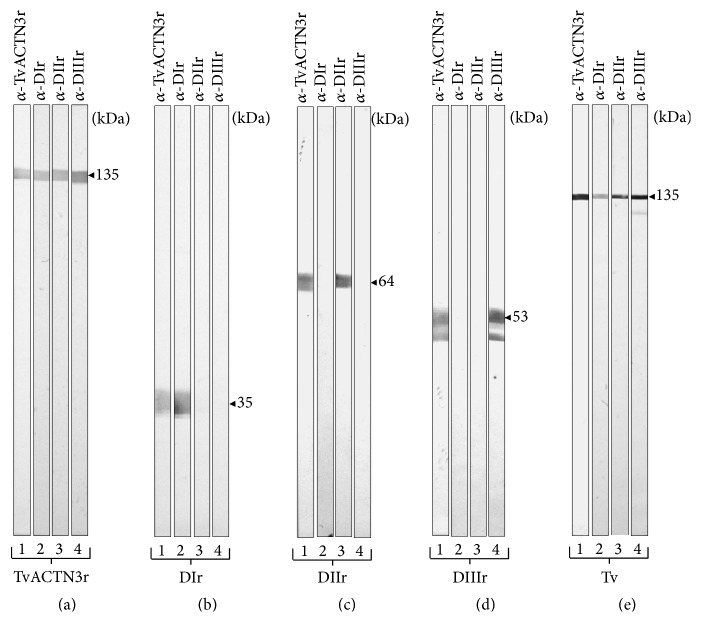
Production of polyclonal antibodies and specific recognition of TvACTN3 domains (DIr, DIIr, and DIIIr) and* T. vaginalis* total protein extracts by the specific antibodies. WB assays using (a) TvACTN3r, (b) DIr, (c) DIIr, (d) DIIIr purified recombinant proteins, and (e) total protein extracts from* T. vaginalis *grown in regular iron conditions used as antigens and transferred onto NC membranes and incubated with *α*-TvACTN3r, *α*-DIr, *α*-DIIr, and *α*-DIIIr polyclonal antibodies (lanes 1–4). kDa, molecular weight markers in kilodaltons (Bio-Rad).
