# Supplementary material for: α-Actinin TvACTN3 of Trichomonas vaginalis Is an RNA-Binding Protein That Could Participate in Its Posttranscriptional Iron Regulatory Mechanism
Source: Biomed Res Int. 2014 Mar 2;2014:424767. doi: 10.1155/2014/424767 (PMC3955661; doi:10.1155/2014/424767)
Supplement: Supplementary file 1 — In this figure we show a multiple alignment of the amino acid deduced sequence of the five actinin genes found in the T. vaginalis genome [8] (TvACTN1, TvACTN2, TvACTN3, TvACTN4, and TvACTN5). These α-actinin proteins have different sizes (609, 931, 1129, 1137, and 1271 amino acid [aa] residues) and different identities compared with TvACTN3 (55.19, 53.25, 22.1, and 15.56% with TvACTN1, TvACTN2, TvACTN4, and TvACTN5, respectively). The most conserved region among them is the N-terminal corresponding to the actin-binding domain. The most divergent region is the central domain that contains the spectrin repeats (Please see Figure 3). [file 424767.f1.docx]

TvACTN3 MSNNR-GLLDDAWEQTQIKVFSRWTAKQLSFKGIPFDNVLEEFADGVKLIQLLEIVSKEP 59

TvACTN2 MSVRREGLLDDAWEKTQIKVFSRWVQKQLLARQIQFETIETDFEDGTKLLNLLEIIGKEP 60

TvACTN1 -----MTTLNKNWEQTQIKVFSRWCNKYCQQAGMKVEDCTQDFEDGVKLITLLEIVGKEP 55

TvACTN4 --------MTRTWEETQINTFTKWMNSYLKQRKLNINDIKTDLCDGTILINLCEILSKKP 52

TvACTN5 --------MPRSWEETQINTFTRWVNSYLRSRDLKVNDLKTDLCDGRLLINLCEILTKQT 52

: **:***:.*::* . : .: :: ** *: * **: *:.

TvACTN3 MKGKWHKQPKMRVQMRENCGMALDFISS-KNIRMVGIGSDDIIDKNKKLTLGLIWTIINK 118

TvACTN2 MPSKWHKQPKMMVQKRENVDLALKYINEVKKIRTVGIGADDIINKNLKLTLGLTWTCINK 120

TvACTN1 MPGKWHKTPKMRIQKLENCDLAIKYISEVKKVRLVGIGSNNIVDHDTKLTLGLIWSVINK 115

TvACTN4 FPSRWHKNPKQKVQMLENCHMAVDFIQNTLKIKLVGIGGEDVLNGTLKLILGLVWSVINK 112

TvACTN5 FPKKWHKTPKLNMQKLENCHMAVDYINKDMQIKLVGIGGDDVNAGNLKLILGLIWSIINK 112

: :*** ** :* ** :*:.:*.. ::: ****.::: ** *** *: ***

TvACTN3 FMIEEISVEEAT------ARDALLLWAKKNTQGYKGVNVTNFTTSWSDGLAFCALINKFR 172

TvACTN2 FMIEEISVEEAT------ARDALLLWAKKNTQGYEHVAVNNFTTSWNTGLAFAALINKFR 174

TvACTN1 FVIEEITVEEAT------ARDALLLWCKKNTQGYEGVDVTNFTSSWTSGLAFCALINHFR 169

TvACTN4 FQVEAIKEDLAEGEQ-ISGRDKLLQWAKESTANHNGVNIVDFSKSWHGGLPFCAILHHYL 171

TvACTN5 YQVEAIQEDFPQTEKKQSGKEKLLNWAKTATADHKGVNITDFSKSWRGGLPLCAIFHHYL 172

: :* * : . .:: ** *.* * .:: * : :*:.** **.:.*:::::

TvACTN3 PNMLDYDSLDQTQQKENCEKAFAACKELGIYVFLDPEDLVGTQPDEKSVVTQVAEFFHFF 232

TvACTN2 PNLLDYSALDYNDHKGACEKAFAACKELGIYVYLDPEDVIDTTPDEKSVVTQVAEFFHFF 234

TvACTN1 PELLDYNALDKANHPENCRLAFEACKQLGITVYLDVEDIADTQPDEKSVVTQVSEFFHFF 229

TvACTN4 PEIVDYDSITPEN---SINEAFLVMNECGINVFLDPQDLEVEAPDEKSVVTQVSELYHFF 228

TvACTN5 PDCINYDSIKPED---SINEAFSFMKRAGVNVFIDPQDLEVAAPDEKSIVTQVSELYHTF 229

*: ::*.:: : . ** :. *: *::* :*: *****:****:*::* *

TvACTN3 AGESKTQAAADKLKRTI----GIQKAIEEEALNYEKQAQECLDVINTEREKLLAQ----- 283

TvACTN2 ASESKIAAMADKIKRTV----AIQKQIDELKNTYIEDAKAAIEKMTVEDEKLKAD----- 285

TvACTN1 ASDTKADQMAEKLKNTV----GIQREIKDMTEAYIQACQAAIAEMDAKSAEIADD----- 280

TvACTN4 KKPENIAAAKRNADRRK----YILATIKKMVNESEGDLHDALVVMSKNIDTLLEDPALNF 284

TvACTN5 KKPEKVEAIHKTLKPIPGFINCTVVNGRNLAAMDKGGKSDPYVIVKINKNGNPHKTEIIK 289

: . . . : : .

TvACTN3 -----DYDQTVPGVKSKLFN------CIKFGRVVRPVIVDKRG--------VAMKTWGQL 324

TvACTN2 -----DYEKTIPGIRGKLAS------VISYNRDIRPEIVDHRA--------KAMRSWAAL 326

TvACTN1 -----SFDKDTKGIKQKLVN------TIAYGKDGRPKIFELKA--------NAVAAYSAL 321

TvACTN4 CPLCAEFFETIEKLYALLKNPKVPADALQQIENLNKQLLEFQEKVKQLDEEKAQKEKEAE 344

TvACTN5 ETLNPDFNQDFTIQFADQKVDSIILECYDWDDHNSHDLIGTAEIQLNQYVFNRVIERDIE 349

.: : :.

TvACTN3 VTKCNSNGRPIPQVKEELLPPTLNLKFEEIEKTASDRRDELTKILEELQAKLINAFDEAA 384

TvACTN2 VTKCKSGNRPIPEIPQGLEPEALTNKFNEIEQTSTTRRDELTQELNDMIKKKVEDFMAKC 386

TvACTN1 QLKCKATKRPMPEIPEEYIEQALNQRFEQLDQQVSDRRSELLGLLN-------------- 367

TvACTN4 ELKQQNNAKEQELQNLKNEKEAKEKELEEVKNEKAAKEQELENVKNEKTAKE-QELENIK 403

TvACTN5 LKKEGGHRKERGTIHFRFILLASLDNTDSEGEDNVVPEENATPVPPIVLNATVIDARDLP 409

* : : . :. : ..:

TvACTN3 NAKIAVCDDINNKAINLTGDLYEQRDALNNYLQQAQEAAG-------------------- 424

TvACTN2 MDIINKCDAIHEEVKTIEGTTAEKKDKVEQKLHEAEDLQP-------------------- 426

TvACTN1 -------------------------DKVKSYVSQAKEVQG-------------------- 382

TvACTN4 NEKEAKEKELENVKNEKAAKEQELENVKNEKAAKEQELEN-------------------- 443

TvACTN5 AMDADGQADPFCILTVNGKGEQFKTRVIKNNLNPVWNHAFNIPINNQFTDTLYVNLIDFD 469

:. :

TvACTN3 TVKELQPQFVELVELRLNNRVKRTVIAVDGEFEQLIATIKRLIEGNKAAIFE--YENKKK 482

TvACTN2 ALAELTPLFQELVELRINTLSSQTDDSVNRHHSQLITYIKHLLEQLNGKLFE--ETNEAR 484

TvACTN1 ALEALD------------------------------------------------------ 388

TvACTN4 VKNEKTAKEQELENIKNEKEAKEKELEEVKNEKTSKEQELENVKNEKAAKEEQLAKMTTD 503

TvACTN5 ETTNNDLIGYNKISLRDLQIGKPEELQLPLRKLHAVRTDRGTVHLMLQAYKPGEEPEIMP 529

TvACTN3 IEEYNQAAQKYVDEVAQLKQDLEAIAGELREQRAANVDKSEEIIQKRNGVSDIRPMFQEL 542

TvACTN2 INEYNALAQPLYDEAIAFKEEVLAISGELRE----------------------------- 515

TvACTN1 -------------------KELDSIEGELEQ----------------------------- 400

TvACTN4 FEQKNNESGNLSSELEQLKQQLAAAQQQNEQLNIMIKAKDNEMNAVIARANEQLQNLNQQ 563

TvACTN5 PKEEEPEVKAFVDCKVISATKLVAMDSNGKSDPYVVLKYNKDGEPQKTEICKKTLNPEWN 589

.: : : ..

TvACTN3 EKQSLHLGIENTPDAVTAMYTACLSQAQDKITQISKQLVDEYNEKAIAIHEKIGDVHKTA 602

TvACTN2 ------------------------------------------------------------

TvACTN1 ------------------------------------------------------------

TvACTN4 KDEELKKKDDEIN----------------------------------------------- 576

TvACTN5 QDFTFTVVQKKTDILYVECWDWDDHNSHDLIG---------------------------- 621

TvACTN3 DSVSGTTQEKKDGALKCQSDLTEIKASIQPTLEEPYQYLQSIKYSNAVKYTPNDLTRDSD 662

TvACTN2 ------------------------------------------------------------

TvACTN1 ------------------------------------------------------------

TvACTN4 ------------------------------------------------------------

TvACTN5 ------------------------------------------------------------

TvACTN3 ITFAFLTTLLNQLEEQLQSESNDARIAAYNELATKYVDIANEFHQKVSTIDGDRATRRNA 722

TvACTN2 --------------------------------------------------------RRTQ 519

TvACTN1 ------------------------------------------------------------

TvACTN4 -------------AIKEAKNSELAQLRSENEQNLAKKDLLLQEKDQEIQKRFREIEDLNN 623

TvACTN5 ---VGEVKIEEFMYDTLVETDVELKKEGGHRKERGTVHLRIFVRTDRTGETDNEMGNTES 678

TvACTN3 YLSAQLELGNKREGLSQLKPEYEALERDTLHIRVNDSPATISKVYANALQIITDKLAEIY 782

TvACTN2 FLAKQAEAPTKREHVNEIDPIFDGLEKDSLHLRVNHSPTEIRNVYAVTLQHIITELNKIF 579

TvACTN1 -------------------------KKEALNAKVD------------------------- 410

TvACTN4 NKEELLKQKDQEIEQMKNLQSQEEKRREAEVSELSQQIEQLKQLQSSGAQEAQNILIETQ 683

TvACTN5 EGEEAPSAQPAETATPIVVHCTVVDAKDLPAMDINGKADPFCQLTVNGKGQEYKTEVVMK 738

:: :.

TvACTN3 NEMVADFDAQVLAIAEKVKVVQN--VELTGTLLELKDKIAQSKAQAQEILPELPTLDAPW 840

TvACTN2 EEMVANFDATAVPIIDGITALVTSSHQIPGDAAAVKAQVEENLASLDGFAEKIQALQDPY 639

TvACTN1 ------------------------------------------------------------

TvACTN4 NKYSAQIAEKDKRIEELENAKSQLEKESEDRNEENKKLLVQCSNLRDEVSSKDKALQESE 743

TvACTN5 NKNPTWNQSFNIPVEDQNKDHLYVTLFDFDKDSDNDLIGYNRIKLRDLPLNTPVEREVEL 798

TvACTN3 EDLCDFNLNYRVKQTPENLRATLEAVIAFLNHQEESNNEKLASEGRESRIFAYNQKAAVS 900

TvACTN2 NELVEFKLNYKVTYTYSDATGELDQARLDLKQIILAKKTFLEEEERKARINNYTVKADEH 699

TvACTN1 ------------------------------------------------------------

TvACTN4 KNLEEAKKEINVIVTKQKQSADDEAVKAAVEGYKEVIAGLKCEYGHGVTKNDTEAFKHYT 803

TvACTN5 KKKHGLRPDRGVAHLILTAYKPGEEPQIEATPVEEPVKSEVPPKAEFLDCTVVSASNLVK 858

TvACTN3 VALA-RELEQKVSNVDGTLPEKQQKLFDIKQEVIDGDE-AAKELTPIFEDLEKDELHLAI 958

TvACTN2 MNEA-HALDGKINSVDGELEPKRQKLYEVREEVNAKKEKAAEELTPIYEDLEKDQLHLEI 758

TvACTN1 ----------KIRSKHG----------------------DVEALAPIYNDIEEAEMHLEI 438

TvACTN4 VAAK-LNIAEGQFSLGRLLEQGRGNIKNERLAFQSYARAQNQNHYAAMNNMAAMLLTGRG 862

TvACTN5 MDKHGLSDPYVVLKVNKDGEPQKTEVVKQNLNPEWNQEFHFTPVDKTKDVLVVECYDWDD 918

. : :

TvACTN3 EDTPDSIAAFFANILSHIDGLVREIDTAIAAEKGLQISEEQLTEFR-------------- 1004

TvACTN2 TSTPASINIFFENLIAHIDTLVKEIDAAIAAAKGLEISEEELNEFK-------------- 804

TvACTN1 DETPSFIESIYNATIAHATTLIREIEAAIAAAKGLEISEEQLASFH-------------- 484

TvACTN4 VDKDEKLAAQKFKVAADNGNVAAQCNYGILLDKGTGVEKNEIDAVRYFRLAANAGHPRAI 922

TvACTN5 HNSHDLIGNAILELAQYAYDIPIEADVELKKEGGHRKDRGTVHLRFTIRK---------- 968

.. : : : : : * .. :

TvACTN3 -------ETFNHFDKDHTNFLQYFELRACLT----------------------------- 1028

TvACTN2 -------DTFKYFDKDKSNSLEYFELKACLT----------------------------- 828

TvACTN1 -------ETFNHFDKDHSKSLQYYELRACLT----------------------------- 508

TvACTN4 NNLAYKLENGSGIDKDHAEATKMFHQAASQGQSVAKFNVSLY-----------ESDQEKV 971

TvACTN5 -------DKTGEPDDEHTTSEEENNKAVAKADPIVLHCTVVDGVELPAMDITGFSDPFVR 1021

:. *.::: : . ..

TvACTN3 -----------------ALGDDISD--------DQAKEVCKKYSATGE-EKLNFDEYVKF 1062

TvACTN2 -----------------ALGEDITD--------DQAKEYCKKYNSKGEGTALEFDDYVRF 863

TvACTN1 -----------------ALGEDFTD--------DQAKETCKKYSASGE-EKLNFDEYVKF 542

TvACTN4 LNEWKLAASQNVPAAKNNIGVTMSDGILLPKNEEEGAELVKTAAEGGS--TAAIFNYSIM 1029

TvACTN5 LTVNGQGKPYTTGIVMRELNPIWNQEFNIPIDNQNKDKLYITCYDWDEDSANDLIGYYRL 1081

:. .: :: : . .. : * :

TvACTN3 MLDHFSKAENADTTAKAFKAIANNNPILTDAQLDQYFKGEEAEYLR--KVLKQVDGGYEF 1120

TvACTN2 MLDHFSKAETTETTMEAFKAIAQNQPVLTDAQLDQYFSAEDAAYLR--SQLKQGENGYEF 921

TvACTN1 MLDHFNKAETNETTMEAFKTIANGNPVLNDAQLDRYFSAEDAAFLR--QELPQVEGGYDF 600

TvACTN4 LMNAKGVEENKKGAARLLKISSDKGYAKAMNNLGVMFIKGEVVKLAPLEGVKMVRKAADE 1089

TvACTN5 PLDDIKVGEPVERECILKKKHALRANRGKIHLKICAFKPGEEPQVSKVPGAHPIKNIKPK 1141

:: * . * : * : :

TvACTN3 AEWVNSIYA--------------------------------------------------- 1129

TvACTN2 ADWVNSLYNQ-------------------------------------------------- 931

TvACTN1 QSWVNKIYA--------------------------------------------------- 609

TvACTN4 LNDPDALFNMGLIFLKGLGVTADPAFAQNYFQKAAKNGNKTAELYLQQ------------ 1137

TvACTN5 ETLLDATVVNARDLVPMDKNGKSDPYVILKLNRNGIPQQTTVVKASLNPDINENFDFTLI 1201

:

TvACTN3 ------------------------------------------------------------

TvACTN2 ------------------------------------------------------------

TvACTN1 ------------------------------------------------------------

TvACTN4 ------------------------------------------------------------

TvACTN5 DPKTDVLLVYCYDWDDHNNHDLIGVGEIPLEGIALDVPVEKQVELKKEGGHRKERGKVNL 1261

TvACTN3 ----------

TvACTN2 ----------

TvACTN1 ----------

TvACTN4 ----------

TvACTN5 KLRLHNSRDG 1271

**Supplementary Figure S1.** Alignment of the amino acid sequences of the four ACTN proteins, as reported in the *T. vaginalis* genome project ([www.trichdb.org](http://www.trichdb.org)) and the TvACTN3 from the CNCD147 isolate. Sequences were aligned using the ClustalW program. (*) In this column the alignment contains identical aa residues in all sequences that are also highlighted in gray. (:) This column of the alignment contains highly conserved aa. (.) This column of the alignment contains similar aa. ( ) This column of the alignment contains different aa or gaps. The IDs of the α-actinins in *T. vaginalis* are TvACTN1: TVAG_156680; TvACTN2: TVAG_190450; TvACTN3: TVAG_239310, GenBank accession number KF280188; TvACTN4: TVAG_247460; and TvACTN5: TVAG_260390.
